# Supplementary material for: Low-dose interleukin-2 in patients with stable ischaemic heart disease and acute coronary syndromes (LILACS): protocol and study rationale for a randomised, double-blind, placebo-controlled, phase I/II clinical trial
Source: BMJ Open. 2018 Sep 17;8(9):e022452. doi: 10.1136/bmjopen-2018-022452 (PMC6144322; doi:10.1136/bmjopen-2018-022452)
Supplement: Supplementary file 2 [file bmjopen-2018-022452supp002.pdf]

Supplementary Docuemnt 1

Risk mitigation table.

Potential Risk	Impact - eligibility criteria	Monitoring criteria	Stopping criteria for individual patients
**Cardiac disorders:**<br>Capillary leak syndrome,<br>Cardiac arrhythmias,<br>Transient ECG changes,<br>Angina,<br>Myocardial infarction,<br>Palpitations,<br>Ventricular hypokinesia	Exclusion criteria include: Cardiogenic shock (as defined by systolic blood pressure <80 mmHg unresponsive to fluids or necessitating catecholamines; hypotension (systolic BP < 100 mmHg and/or diastolic BP < 50 mmHg); uncontrolled hypertension (>160/100mmHg); history of recurrent syncope with relevant history suggestive of arrhythmic syncope (e.g. bifascicular block, sinus bradycardia < 40 bpm in the absence of sinoatrial block or medications, preexcited QRS complex, ST segment elevation leads V1 through V3 [Brugada syndrome], negative T wave in the right precordial leads and epsilon wave [arrhythmogenic right ventricular dysplasia/cardiomyopathy], prolonged QT > 450 msecs (or > 480 msecs for patients with bundle branch block); known heart failure with impaired Left ventricular, function (echocardiographically assessed Left ventricular ejection fraction, LVEF < 45% Part A, LVEF < 35% Part B); severe congestive heart failure and/or pulmonary oedema on presentation; ST elevation Myocardial infarction;	On a day-to-day basis vital signs (temperature, blood pressure, heart rate, respiratory rate) will be assessed pre-dosing and every 30 mins (approximately) for 1h post-dosing; 12 lead ECG with QTcB measurement will be performed pre-dosing and approximately 15, 30 mins and at 1 h post-dosing;<br>Continuous cardiac telemetry will be applied during the trial visits for a minimum of 2 hours and up to 6.5 hours.<br>Cardiac biomarkers (TnI and BNP) will be taken prior to dosing (V2) and at the end of active treatment period (V7 and V8).<br>Baseline and post dose echocardiogram will be performed (V1 and V8)	Treatment with the trial drug will be discontinued if:<br>QTcB > 500 msecs (or > 530 msecs if baseline QTcB = 450-480 msecs) OR QTcB change from baseline > 60 msecs (based on an average of triplicate ECGs);<br>New or worsening angina in stable patients (Part A), Worsening angina in ACS patients (Part B);<br>Acute Pulmonary oedema or congestive heart failure;<br>BP stopping criteria: symptomatic systolic BP < 90 and/or diastolic BP < 60 OR persistent symptomatic systolic BP 80-90 mmHg for > 15 mins; OR severe hypertension (as defined by BP > 180/120 mmHg);<br>STEMI occurrence;<br>Atrial fibrillation with rapid ventricular response > 150/min, supraventricular tachycardia or bradycardia that requires treatment or is recurrent or persistent;<br>Sustained ventricular tachycardia or ventricular fibrillation
**Kidney injury or impaired renal function:**<br>Oliguria,<br>Raised serum urea,<br>Raised serum creatinine,<br>Haematuria,<br>Renal failure,<br>Anuria	Part A: Patients with Acute kidney injury (doubling of the serum creatinine from baseline) and/or CKD more than Stage 3B (eGFR = 30-45 ml/min/1.73m^2) will be excluded<br>Part B: Patients with Acute renal impairment at screening (Creatinine clearance [Cockcroft-Gault] <45ml/min) will be excluded	Part A:Kidney function parameters (including serum creatinine, BUN, electrolytes , calcium and eGFR) will be assessed at all visits)<br>Part B:Kidney function parameters (including serum creatinine, BUN, electrolytes and calcium) will be assessed at all visits)	Any patient who develops doubling of creatinine during the trial will be withdrawn from the trial.
**Risk associated with subcutaneous injection of IL-2:**<br>Injection site reaction, pain, inflammation,<br>Mucositis,<br>Injection site nodule,<br>Hypothermia,<br>Injection site necrosis,<br>Erythema,	Patients with a history of known allergy or skin hypersensitivity to IL-2 or any of its excipients will be excluded. History of drug induced Stevens Johnson syndrome, Drug reaction with eosinophilia and systemic symptoms (DRESS syndrome) or toxic epidermal necrolysis.	Injection sites will be examined at each visit and the patients will be assessed for AEs which may be linked to IL-2 related hypersensitivity reactions (erythema, pruritus, angioedema or generalized urticaria).	The investigators should stop the dosing of any patients with any systemic hypersensitivity reaction which cannot be attributed to an identifiable cause. Patients who are dosed and then go on to have a contrast reaction during

Pruritus,<br>Urticaria<br>Malaise, asthenia and fatigue,<br>Pain,<br>Oedema,<br>Weight gain/loss,<br>contrast allergy	Patients with a history of contrast allergy will be excluded from the study in Part B.		this study will be withdrawn from the study
**Infections:**<br>Sepsis<br>Fever with/without chills,	Exclusion criteria include: active infection requiring antibiotic treatment; leukopenia (WBC < 3.3 x 10^3/μL); uncontrolled diabetes (HbA1c > 64 mmol/mol Part A only); current infection possibly related to recent or ongoing immunosuppressive treatment; any oral or intravenous immunosuppressive treatment (including steroids or disease modifying agents such as azathioprine, interferon-alpha, cyclophosphamide, mycophenolate [other immunosuppressive therapies should be discussed with PI]; known HIV infection; known chronic active hepatitis B or C<br><br>Patients with recent infections will only be included when deemed clinically stable by the investigators (when the infection is resolved).	On a day-to-day basis vital signs (temperature, blood pressure, heart rate, respiratory rate) will be assessed pre-dosing and every 30 mins (approximately) for 1h post-dosing<br>Inflammation markers, including WBC + differential as well as CRP will be assessed at baseline and during the treatment period on a daily basis<br>If a patient is found to have pyrexia > 38.5ºC (either in the unit or at home) on 2 separate occasions, diagnostic evaluation (CXR, urine dipstick, blood and urine cultures as directed by symptoms) will be initiated.	If an infection is confirmed clinically with a positive microbiological test, the trial medication will be discontinued.
**Gastrointestinal adverse events:**<br>Nausea with/without vomiting,<br>Diarrhoea,<br>Stomatitis,<br>Dysphagia,<br>Dyspepsia,<br>Constipation,<br>GI bleeding (including rectal haemorrhage, haematemesis),<br>Ascites,<br>Cheilitis,<br>Gastritis,<br>Pancreatitis,<br>Intestinal obstruction,<br>GI perforation,<br>Elevation of hepatic transaminases/ alkaline phosphatase/ lactic dehydrogenase,<br>Hyperbilirubinaemia,<br>Hepatomegaly/ Hepatosplenomegaly<br>Cholecystitis,<br>Liver failure	Exclusion criteria include: known active bleeding (including GI bleeding) or bleeding diatheses; known hepatic failure and/or abnormal LFTs (ALT > 2 x ULN) at baseline; elevated total bilirubin (TBL > 1.5 x ULN) and/or Alkaline Phosphatase levels (ALP > 1.5 x ULN) at baseline; history of chronic active hepatitis B or C;	On a day-to-day basis vital signs, including blood pressure and heart rate will be assessed pre-dosing and every 30 mins (approximately) for 1h post-dosing<br>A daily clinical assessment, including abdominal, skin and mucosal examination will be performed as part of the physical examination.<br>Haemoglobin, haematocrit, platelet counts, BUN, blood glucose, LFTs, TBL and ALP will be assessed at all study visits.	IL-2 treatment should be stopped if the patients show signs suggestive of hepatic failure including encephalopathy, increasing ascites, signs of coagulopathy, liver pain and/or tenderness on palpation, hypoglycaemia presumed to be secondary to liver failure, active GI bleeding. Withdrawal also if ALT >3 ULN
**Neurological events:**<br>Dizziness<br>Headaches<br>Paraesthesia<br>Neuropathy<br>Syncope<br>Speech disorders	Patients with history of recurrent epileptic seizures in the previous 4 years, repetitive or difficult to control seizures, coma or toxic psychosis lasting >48 hours will be excluded.	Changes to the mental status of the trial patients will be monitored during the physical examination for any signs, including moderate confusion or agitation.	The drug will be discontinued in patients who develop seizure activity, coma, severe lethargy or somnolence.

Taste loss<br>Lethargy<br>Coma<br>Convulsions<br>Paralysis<br>Myasthenia<br>Intracranial haemorrhage<br>Cerebrovascular accident<br>Leukoencephalopathy			
**Respiratory events:**<br>Respiratory tract infection,<br>Cough,<br>Dyspnoea,<br>Pulmonary oedema,<br>Pleural effusions,<br>Hypoxia,<br>Haemoptysis,<br>Epistaxis,<br>Nasal congestion,<br>Rhinitis,<br>Pulmonary embolism,<br>Adult respiratory distress syndrome	Patients with a history of underlying respiratory failure, requiring intubation for > 72 hours will be excluded.	Respiratory rates and spO2 will be routinely monitored.	The drug will be discontinued in patients at risk of respiratory insufficiency requiring intubation.
**Embryofetal lethality**<br>Embryofetal studies in rats have shown embryolethality in the presence of maternal toxicity.	Lactating or pregnant female patients will be excluded.	Pregnancy tests for females of child-bearing potential will be performed at screening visit and visit 2 (Part A only) prior to dosing.	Patients who become pregnant during the trial will be withdrawn and followed up appropriately.
