# Supplementary material for: Low-dose interleukin-2 in patients with stable ischaemic heart disease and acute coronary syndromes (LILACS): protocol and study rationale for a randomised, double-blind, placebo-controlled, phase I/II clinical trial
Source: BMJ Open. 2018 Sep 17;8(9):e022452. doi: 10.1136/bmjopen-2018-022452 (PMC6144322; doi:10.1136/bmjopen-2018-022452)
Supplement: Supplementary file 1 [file bmjopen-2018-022452supp001.pdf]

Trial Title: Low dose interleukin-2 in patients with stable ischaemic heart disease and acute coronary syndromes (LILACS)

EudraCT No.: 2014-004979-23

IRAS No.: 168647

Trial Sponsor: Cambridge University Hospitals NHS Foundation Trust and University of Cambridge.

Data Monitoring Committee (DMC) Charter

Version 1.1 date 09 NOV 2017
(Developed from DAMOCLES DMC Charter Template v1. February 2005)

Authorised by:

Name:	Role:
Signature:	Date:

Prepared by:

Name:	Role:
Signature:	Date:

Table of Contents

CONTENT 3
CHARTER DETAILS 3
1. Introduction 3
Trial Intervention 3
Part A 3
Part B 3
2. Roles and responsibilities 4
3. Before or early in the trial 5
4. Composition 6
5. Relationships 7
6. Organisation of DMC meetings 7
7. Trial documentation and procedures to ensure confidentiality and proper communication ... 8
8. Decision making 9
9. Reporting 10
10. After the trial 11
Figures and appendices 12
Annex 1: Agreement and potential competing interests form 14
Annex 2: Agreement and confidentiality agreement for observers 15
Annex 3: Suggested report from DMC to TMG where no recommendations are being made .. 16
Annex 4: LILACS Contacts 17
Annex 5: Summary of changes from previous version 18

CONTENT	CHARTER DETAILS
**1. INTRODUCTION**	
Name of trial	Low dose interleukin-2 (IL-2) in patients with stable ischaemic heart disease and acute coronary syndromes (LILACS)
Objectives of trial, including interventions being investigated	**TRIAL INTERVENTION**<br>Low-dose IL-2 (aldesleukin)<br><br>**PART A**<br>PRIMARY OBJECTIVES<br>1. Is the administration of IL-2 safe and tolerable in patients with stable ischaemic heart disease?<br><br>Exploratory objectives:<br>1. Does administration of low-dose IL-2 in stable ischaemic heart disease patients result in an increase of circulating Treg levels?<br>2. Does administration of low-dose IL-2 in stable ischaemic heart disease patients result in changes in circulating cardiac biomarkers (including hs-CRP, TnI, IL-6, BNP)?<br>3. Does administration of low-dose IL-2 in stable ischaemic heart disease patients result in changes in lymphocyte subsets?<br>4. Does administration of low-dose IL-2 in stable ischaemic heart disease patients result in changes in circulating IL-2 levels?<br><br>**PART B**<br>Primary objectives<br>1. Does low-dose IL-2 administration in patients with acute coronary syndromes result in an increase mean circulating Treg levels by ≥75% (placebo corrected)?<br>2. Is the administration of IL-2 safe and tolerable in patients with ACS?<br><br>Secondary objectives<br>1. Does administration of low-dose IL-2 in ACS patients result in changes in circulating cardiac biomarkers (including hs-CRP, TnI, IL-6, BNP)?<br>2. Does administration of low-dose IL-2 in ACS patients result in changes in lymphocyte subsets?<br>3. Does administration of low-dose IL-2 in ACS patients result in changes in circulating IL-2 levels?
Outline of scope of charter	The purpose of this document is to describe the membership, terms of reference, roles, responsibilities, authority and decision making of the DMC for the LILACS trial. This includes the timing of meetings, methods of providing information to and from the DMC, frequency and format of meetings, statistical issues and relationships with other committees.

CONTENT	CHARTER DETAILS
**2. Roles and responsibilities**	
A broad statement of the aims of the committee	To protect and serve LILACS trial patients regarding safety and to assist and advise the Chief Investigator and Trial Management Group (TMG) so as to protect the validity and credibility of the trial.<br>To safeguard the interests of LILACS patients, assess the safety and efficacy of the interventions during the trial, and monitor the overall conduct of the LILACS trial.
Terms of reference	The DMC should receive and review the progress and accruing data of the LILACS trial and provide advice on the conduct of the trial to the TMG.<br>The DMC should inform the Chair of the TMG if, in their view:<br>(i) the results are likely to convince a broad range of clinicians, including those supporting the trial and the general clinical community, that one trial arm, or a subset of trial population, is clearly indicated or contraindicated, and there was a reasonable expectation that this new evidence would materially influence patient management; **or**<br>(ii) it becomes evident that no clear outcome would be obtained.
Specific roles of DMC	The DMC will review the trial data after completion of Part A of the trial and will provide recommendation for the dose to be used in group B1 (Part B of the trial). The review of the trial's progress will include data quality, and main endpoints including safety data.<br>In addition, a DMC meeting might be triggered when:<br>Two patients within a trial group experience any combination of: a serious adverse event (SAE) defined as possibly, probably or definitely related to the trial drug (i.e. it is a SAR), an adverse event that is severe and at least possibly related to the trial drug, or any of the objective stopping criteria detailed in the protocol (Any further single instances of the events outlined above for the same group will trigger a DMC safety review). See current protocol for full details of a triggered DMC meeting.<br><br>Specific roles of the DMC include:<br>• assess data quality, including completeness and accuracy (and by so doing encourage collection of high quality data)<br>• monitor participant and investigator compliance with the protocol<br>• monitor evidence for treatment differences in the main efficacy endpoints<br>• monitor evidence for treatment harm (eg toxicity data,

CONTENT	CHARTER DETAILS
	SAEs, deaths)<br>• review all reports of suspected unexpected serious adverse reactions (SUSARs) provided by the trial team<br>• decide whether to recommend that the trial continues to recruit participants or whether recruitment should be terminated either for everyone or for some treatment groups and/or some participant subgroups<br>• suggest additional data analyses<br>• advise on protocol modifications suggested by the TMG (eg inclusion criteria, trial endpoints, or sample size)<br>• monitor continuing appropriateness of patient information<br>• monitor compliance with previous DMC recommendations<br>• consider the ethical implications of any recommendations made by the DMC<br>• assess the impact and relevance of external evidence<br>• maintain confidentiality of all trial information that is not in the public domain<br>• protect validity and scientific credibility of the trial
**3. BEFORE OR EARLY IN THE TRIAL**	
Whether the DMC will have input into the protocol	All potential DMC members should have sight of the protocol/outline before agreeing to join the committee. Before recruitment begins the trial will have undergone review by the funder/sponsor (eg peer review for public sector trials), scrutiny by other trial committees, a research ethics committee (REC), Medicines and Healthcare products Regulatory Agency (MHRA) and Health Research Authority. Therefore, if a potential DMC member has major reservations about the trial (eg the protocol or the logistics) they should report these to the CI or trial coordinating team and may decide not to accept the invitation to join. DMC members should be independent and constructively critical of the ongoing trial, but also supportive of aims and methods of the trial.
Whether the DMC will meet before the start of the trial	It is recommended that, if possible, the DMC meets before the trial starts or early in the course of the trial, to discuss the protocol, the trial, any analysis plan, future meetings, and to have the opportunity to clarify any aspects with the CI(s) and coordinating team. The DMC should meet within one year of recruitment commencing.<br>Consideration should be given to an initial "dummy" report, including the use of shell (empty) tables, to familiarise the DMC members with the format that will be used in the reports.
Any issues specific to the disease under study	The use of IL-2 in cardiovascular patients is currently contraindicated. Part A patients have stable ischaemic heart disease and Part B patients have suffered an Acute

CONTENT	CHARTER DETAILS
	Coronary Syndrome (ACS).
Any specific regulatory issues	The DMC should be aware of any regulatory implications of their recommendations.
Whether members of the DMC will have a contract	DMC member will not formally sign a contract but should formally register their assent to join the group by confirming (1) that they agree to be on the DMC and (2) that they agree with the contents of this Charter. Any competing interests should be declared at the same time. Members should complete and return the form in Annex 1. All members and observers attending any part of the meeting should sign a confidentiality agreement on the first occasion they attend all or part of a meeting (Annex 2).
**4. Composition**	
Membership and size of the DMC	Membership should consist of a small number of members, who include at least one clinician experienced in the clinical area. Additional members experienced in clinical trials should reflect the other specialities involved in the trial. In the case of intergroup trials or trials with international collaboration consideration should be given to overseas members.<br><br>The members should not be involved with the trial in any other way or have some competing interest that could impact on the trial. Any competing interests, both real and potential, should be declared. Although members may well be able to act objectively despite such connections, complete disclosure enhances credibility. A short competing interest form should be completed and returned by the DMC members to the trial coordinating team (Annex 1).<br><br>The members of the DMC for this trial are:<br>(1)<br>(2)<br>(3)<br>(4)
The Chair, how they are chosen and the Chair's role	The Chair should have previous experience of serving on DMCs and experience of chairing meetings, and should be able to facilitate and summarise discussions. The Chair is usually chosen by the CI or TMG or sometimes by the DMC members themselves. The Chair is expected to facilitate and summarise discussions.
The responsibilities of the trial statistician	The trial statistician will have the overall responsibility for producing the report to the DMC and will participate in DMC meetings, guiding the DMC through the report, participating in DMC discussions and, on some occasions, taking notes.
The responsibilities of the trial coordinating team	The trial coordinator/or project manager may help the trial statistician to produce the non-confidential sections of the

CONTENT	CHARTER DETAILS
	DMC report. The trial coordinator/or project manager may attend open sessions of the meeting.
The responsibilities of the CI and other members of the TMG	The CI, may be asked, and should be available, to attend open sessions of the DMC meeting. The other TMG members will not usually be expected to attend but can attend open sessions when necessary (See Section 6. Organisation of DMC Meetings).
**5. Relationships**	
Relationships with CI(s), other trial committees (TMG), sponsor and regulatory bodies	A diagram is included in this charter (Figure 2) to illustrate the relationships between the trial committees and the sponsor.
Clarification of whether the DMC are advisory (make recommendations) or executive (make decisions)	The TMG will be responsible for the overall supervision of the trial progress, including the choice of doses to give subsequent cohorts of patients. The TMG will meet between trial cohorts within Part A and Part B of the trial and will make executive decisions about the trial during these meetings.<br><br>The DMC will meet between Part A and Part B of the trial to review all data collected in Part A and will determine whether it is safe to progress to Part B of the trial. In addition a DMC meeting may be triggered for safety reasons which are defined in the trial protocol and under 'specific roles of the DMC' in section 2. Under these circumstances the DMC will make executive decisions about the trial.<br><br>If a DMC meeting is convened for reasons other than those described above then their role will be in an advisory capacity to the TMG.
Payments to DMC members	Members will be reimbursed for travel and accommodation where required. No other payments or rewards are given.<br>DMC members should not use interim results to inform trading in pharmaceutical shares, and careful consideration should be given to trading in stock of companies with competing products.
**6. Organisation of DMC meetings**	
Expected frequency of DMC meetings	The exact frequency of meetings will depend upon any statistical plans specified and otherwise on trial events. The wishes of the DMC and needs of the trial coordinating team will be considered when planning each meeting. The DMC should meet at least yearly.<br>An unplanned DMC meeting may be called by the Chair or requested by the TMG if there is an emergency concern on the safety of participants.
Whether meetings will be face-to-face or by teleconference	The first meeting should ideally be face-to-face to facilitate full discussion and allow members to get to know each

CONTENT	CHARTER DETAILS
	other. If this not possible a video meeting (e.g via skype or GoTomeeting) will be arranged. It is intended that all subsequent meetings should be face-to-face if possible, with teleconference as a second option.
How DMC meetings will be organised, especially regarding open and closed sessions, including who will be present in each session	DMC meetings may contain a mixture of open and closed sessions.<br><br>Closed sessions:<br>Only DMC members and others whom they specifically invite, e.g. the trial statistician, are present in closed sessions.<br>Open sessions:<br>All those attending the closed session may be joined by the CI(s), other members of the trial coordinating team, and sometimes also representatives of the sponsor, funder, or regulator, as relevant.<br><br>Suggested DMC meeting format:<br>1. Open session: Introduction and any "open" parts of the report<br>2. Closed session: DMC discussion of "closed" parts of the report<br>3. Closed session: DMC members private meeting<br>4. Open session: Discussion with other attendees on any matters arising from the previous session(s).<br>5. Closed session: extra closed session as required
**7. TRIAL DOCUMENTATION AND PROCEDURES TO ENSURE CONFIDENTIALITY AND PROPER COMMUNICATION**	
Intended content of material to be available in open sessions	Open sessions: Accumulating information relating to recruitment and data quality (eg data return rates, sample collection) will be presented. Toxicity details based on pooled data will be presented and total numbers of events for the primary outcome measure and other outcome measures may be presented, at the discretion of the DMC.
Intended content of material to be available in closed sessions	Closed sessions: In addition to all the material available in the open session, the closed session material will include efficacy and safety data by treatment group.
Will the DMC be blinded to the treatment allocation	The DMC will not be blinded to the treatment allocation.
The people who will see the accumulating data and interim analysis	The confidential accumulating data and interim analysis by treatment allocation will be seen by the DMC members and the trial statistician(s).<br>DMC members do **not** have the right to share confidential information with anyone outside the DMC, including the CI.
Responsibility for identifying and circulating external evidence (eg from other trials/	Identification and circulation of external evidence (eg from other trials/ systematic reviews) is not the responsibility of the DMC members. The CI, TMG and the trial coordinating team will collate any such information for the presentation

CONTENT	CHARTER DETAILS
systematic reviews)	in an open session.
To whom the DMC will communicate the decisions/ recommendations that are reached	The DMC should report its decisions / recommendations in writing to the CI and TMG chair. This should be copied to the trial statistician (or trial coordinator) and if possible should be sent via the trial statistician (or trial coordinator) in time for consideration at a TMG meeting where necessary. If the trial is to continue largely unchanged then it is often useful for the report from the DMC to include a summary paragraph suitable for trial promotion purposes. (See Annex 3.)<br>In its communications, the DMC should be careful not to relay any unnecessary information to the TMG.
Whether reports to the DMC be available before the meeting or only at/during the meeting	For planned DMC meetings it is usually helpful for the DMC to receive the report at least 2 weeks before any meetings. For unplanned meetings it may be preferable for all papers to be brought to face-to-face meetings by the trial statistician; time would then be needed for DMC members to assimilate the data/report.
What will happen to the confidential papers after the meeting	The DMC members should store the papers safely after each meeting so they may check the next report against them. After the trial is reported, the DMC members should destroy all interim reports. A copy of all the reports will be held at the Cambridge Clinical Trials Unit.
**8. DECISION MAKING**	
What decisions/recommendations will be open to the DMC	Possible decisions/recommendations could include:<br>• No action needed, trial continues as planned<br>• Early stopping due, for example, to clear benefit or harm of a treatment, futility, or external evidence<br>• Stopping recruitment within a subgroup<br>• Extending recruitment or extending follow-up<br>• Sanctioning and/or proposing protocol changes
The role of formal statistical methods, specifically which methods will be used and whether they will be used as guidelines or rules	Interim analyses are scheduled to occur between each group of patients in Part A and Part B of the trial. A report will be generated by the trial statistician that will be reviewed by the TMG who will make decisions about the dose to be used in the next cohort of the trial.<br><br>In addition the DMC will meet to review the safety after Part A of the trial and will decide whether it is safe to progress to Part B of the trial. The minimum dataset required for review between trial groups will be:<br>• All adverse events/adverse reactions<br>• All ECG, blood test results, physical examination reports, echo reports, telemetry summaries and observations up to the V7 time point at a minimum (if not V8)

CONTENT	CHARTER DETAILS
	• T cell subsets including Tregs and Teffs<br>IL-2 levels would be desirable however not mandatory
How decisions or recommendations will be reached within the DMC	The DMC chair should summarise discussions and encourage consensus; it may be best for the Chair to give their own opinion last. It is important that the implications (e.g. ethical, statistical, practical, financial) for the trial be considered before any recommendation is made<br>It is recommended that every effort should be made for the DMC to reach a unanimous decision. If the DMC cannot achieve this, a vote may be taken, although details of the vote should not be routinely included in the report to the TMG as these may inappropriately convey information about the state of the trial data.
Can DMC members who cannot attend the meeting input	If the report is circulated before the meeting, DMC members who will not be able to attend the meeting may pass comments to the DMC Chair for consideration during the discussions.
What happens to members who do not attend meetings	If a member does not attend a meeting, it should be ensured that the member is available for the next meeting. If a member does not attend a second meeting, they should be asked if they wish to remain part of the DMC.
**9. REPORTING**	
To whom will the DMC report their recommendations/decisions, and in what form	This will be a letter to the CI and TMG chair delivered within 3 weeks for planned meetings e.g. for decisions about progression to part B of the trial, and as promptly as possible following unplanned/triggered meetings.<br>A copy of the DMC recommendations/decision letters will be stored in the trial master file.
Whether minutes of the meeting be made and, if so, by whom and where they will be kept	Minutes of the open session will be recorded by a member of the CCTU. Minutes will be finalised upon signature of the chairperson and maintained by the sponsors in accordance with applicable statutory regulations.<br>The minutes of the closed sessions will be recorded by a DMC designee. Minutes from the closed session will be recorded separately from the minutes of the open session and stored securely by the sponsor. Closed session minutes, finalised by signature of the chairperson, will be maintained in confidence and retained until discarded in accordance with applicable statutory regulation.<br>Following each meeting, a report separate from the minutes of the open and closed sessions will be sent to the sponsor/TMG describing the DMC recommendations and rationale for such.
What will be done if there is disagreement between the DMC and the body to which it reports	If the DMC has serious problems or concerns with the TMG decision or vice versa a meeting of these groups should be held. The information to be shown would depend upon the action proposed and the DMC's concerns. Depending on the reason for the disagreement confidential data will often

CONTENT	CHARTER DETAILS
	have to be revealed to all those attending such a meeting. The meeting should be chaired by a senior member of the CCTU or an external expert who is not directly involved with the trial.
**10. After the trial**	
Publication of results	At the end of the trial there may be a meeting to allow the DMC to discuss the final data with the key members of TMG and give advice about data interpretation.<br>The DMC may wish to see a statement that the trial results will be published in a correct and timely manner.
The information about the DMC that will be included in published trial reports	DMC members should be named and their affiliations listed in the main report, unless they explicitly request otherwise. A brief summary of the timings and conclusions of DMC meetings should be included in the body of this paper.
Whether the DMC will have the opportunity to approve publications, especially with respect to reporting of any DMC recommendation regarding termination of a trial	The DMC will be given the opportunity to read and comment on publications before submission.
Any constraints on DMC members divulging information about their deliberations after the trial has been published	The DMC may discuss issues from their involvement in the trial 12 months after the primary trial results have been published, or sooner with permission from the TMG.

FIGURES AND APPENDICES

Figure 1. Trial Flow chart

Figure 2. Relationship of trial committees, including DMC and TMG

Table 1. List of terms

Annex 1: Agreement and potential competing interests form

Annex 2: Agreement and confidentiality agreement for observers

Annex 3: Suggested report from DMC to TMG where no recommendations are being made

Annex 4: Trial Contacts

Annex 5: Summary of changes from previous versions

Figure 1. Trial Flow chart

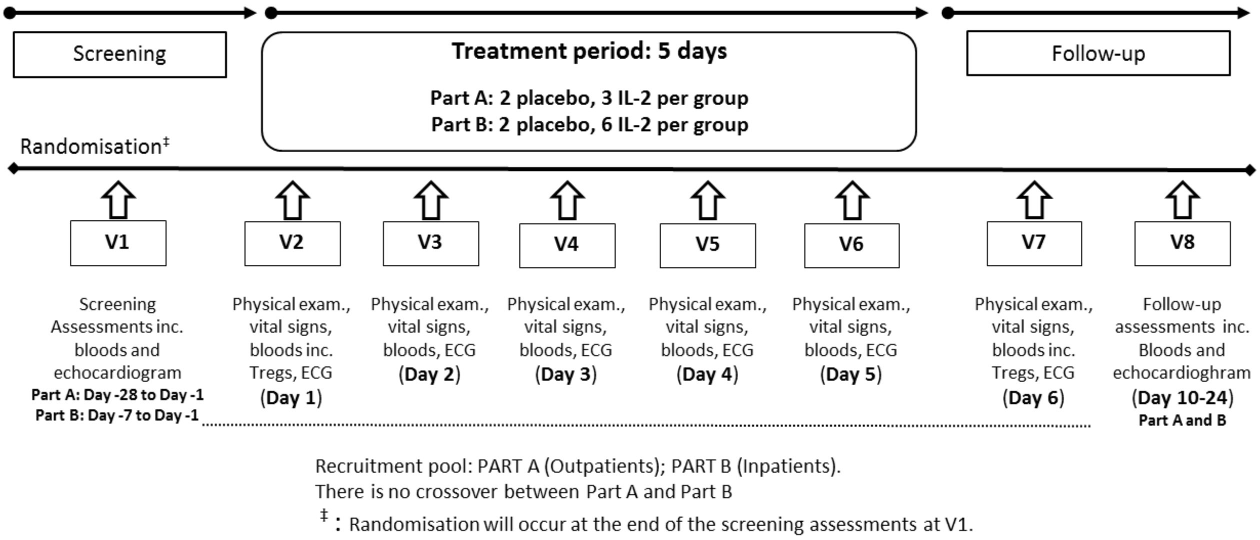

Statement of design

This is a repeat-dose, double blind, placebo-controlled, adaptive trial. There are 2 parts of the trial: Part A and Part B, Part B will only begin once Part A has completed. Part A will include in patients with a history of stable ischaemic heart disease, who will be recruited on an outpatient basis, and, Part B will include patients with ACS will be enrolled from an inpatient setting.

Figure 2. Relationship of trial committees, including, DMC and TMG

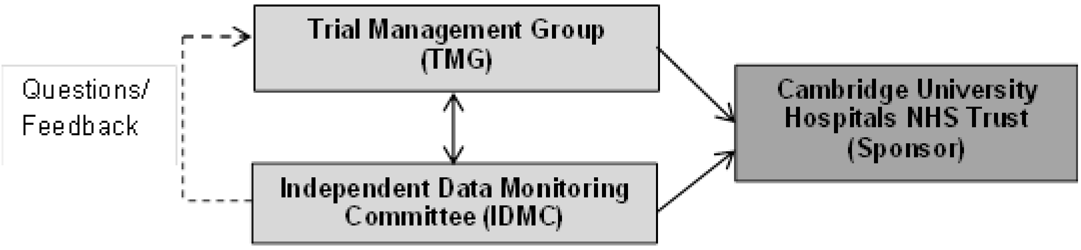

Table 1. List of Terms

Term	Definition
ACS	Acute Coronary Syndrome
AE	Adverse Event
CCTU	Cambridge Clinical Trials Unit
CI	Chief Investigator
CRF	Case Report Form
CTC	Clinical Trials Coordinator
CTIMP	Clinical Trial of an Investigational Medicinal Product
CTM	Clinical Trials Monitor
DM	Data Manager
DMC	Data Monitoring Committee
DMP	Data Management Plan
ID	Identity
HRA	Health Research Authority
IL-2	Interleukin-2
ISF	Investigator Site File
MACRO	Clinical data Management System
MHRA	Medicines Healthcare products Regulatory Agency
PI	Principal Investigator
PID	Patient Identifiable Data
REC	Research Ethics Committee
SAE	Serious Adverse Event
SAR	Serious Adverse Reaction
SDV	Source Data Verification
SUSAR	Suspected Unexpected Serious Adverse Event
Teffs	Effector T cells
TMF	Trial Master File
TMG	Trial Management Group
Tregs	Regulatory T cells

ANNEX 1: AGREEMENT AND POTENTIAL COMPETING INTERESTS FORM

LILACS

Please complete the following document and return to the LILACS Trial Co-ordinator.

(please initial box to agree)

☐ I have read and understood the DMC Charter version 1.0, dated 08 May 2017

☐ I agree to join the DMC for this trial

☐ I agree to treat all sensitive trial data and discussions confidentially

The avoidance of any perception that members of a DMC may be biased in some fashion is important for the credibility of the decisions made by the DMC and for the integrity of the trial.

Possible competing interest should be disclosed via the Cambridge Clinical Trials Unit (CCTU). In many cases simple disclosure up front should be sufficient. Otherwise, the (potential) DMC member should remove the conflict or stop participating in the DMC. **Table 1** lists potential competing interests.

☐ **No,** I have no competing interests to declare
☐ **Yes,** I have competing interests to declare (please detail below)

Please provide details of any competing interests:

____

____

____

Name: ____________________________

Signed: ___________________________ Date: ______________

Table 1: Potential competing interests

- Stock ownership in any commercial companies involved
- Stock transaction in any commercial company involved (if previously holding stock)
- Consulting arrangements with the Sponsor (including Chief Investigator for other Cambridge Clinical Trials Unit)
- Frequent speaking engagements on behalf of the intervention
- Career tied up in a product or technique assessed by trial
- Hands-on participation in the trial
- Involvement in the running of the trial
- Emotional involvement in the trial
- Intellectual conflict e.g. strong prior belief in the trial's experimental arm
- Involvement in regulatory issues relevant to the trial procedures
- Investment (financial or intellectual) or career tied up in competing products
- Involvement in the publication in the form of authorship

ANNEX 2: AGREEMENT AND CONFIDENTIALITY AGREEMENT FOR OBSERVERS

LILACS

Please complete the following document and return to the LILACS Co-ordinator.

(please initial box to agree)

	I have received a copy of the DMC Charter version 1.1, dated 09 Nov 2017
	I agree to attend the DMC meeting on ___/____/______
	I agree to treat as confidential any sensitive trial information gained during this meeting unless explicitly permitted

Name: ____________________________

Signed: ___________________________ Date: ______________

ANNEX 3: SUGGESTED REPORT FROM DMC TO TMG WHERE NO RECOMMENDATIONS ARE BEING MADE

[Insert date]

To: Chair of Trial Management Group
Via: Trial statistician or Trial co-ordinator

Dear *[Chair of Trial Management Group]*

The Independent Data Monitoring Committee (DMC) for the LILACS met on *[meeting date]* to review its progress and interim accumulating data. *[List members] attended* the meeting and reviewed the report.

The DMC should like to congratulate the investigators and trial team on the running of the trial and its recruitment, data quality and follow-up. The trial question remains important and, on the basis of the data reviewed at this stage, we recommend continuation of the trial according to the current version of the protocol *[specify protocol version number and date]* with no changes.

We shall next review the progress and data *[provide approximate timing]*

Yours sincerely,

Name of the Chair,

On behalf of the DMC (all members listed below)

DMC members:
(1)
(2)
(3)
(4)

ANNEX 4: LILACS CONTACTS

ANNEX 5: SUMMARY OF CHANGES FROM PREVIOUS VERSION

Version 1.1 Dated 9 Nov 2017: Figure 1 updated and name of Data Manager added.
