# Supplementary material for: Low-dose interleukin-2 in patients with stable ischaemic heart disease and acute coronary syndromes (LILACS): protocol and study rationale for a randomised, double-blind, placebo-controlled, phase I/II clinical trial
Source: BMJ Open. 2018 Sep 17;8(9):e022452. doi: 10.1136/bmjopen-2018-022452 (PMC6144322; doi:10.1136/bmjopen-2018-022452)
Supplement: Author's manuscript [file bmjopen-2018-022452.draft_revisions.pdf]

BMJ Open is committed to open peer review. As part of this commitment we make the peer review history of every article we publish publicly available.

When an article is published we post the peer reviewers' comments and the authors' responses online. We also post the versions of the paper that were used during peer review. These are the versions that the peer review comments apply to.

The versions of the paper that follow are the versions that were submitted during the peer review process. They are not the versions of record or the final published versions. They should not be cited or distributed as the published version of this manuscript.

BMJ Open is an open access journal and the full, final, typeset and author-corrected version of record of the manuscript is available on our site with no access controls, subscription charges or pay-per-view fees (http://bmjopen.bmj.com).

If you have any questions on BMJ Open's open peer review process please email info.bmjopen@bmj.com

BMJ Open

Low dose interleukin-2 in patients with stable ischaemic heart disease and acute coronary syndromes (LILACS): protocol and study rationale for a randomised, double-blind, placebo controlled, phase I/II clinical trial

Journal:	*BMJ Open*
Manuscript ID	bmjopen-2018-022452
Article Type:	Protocol
Date Submitted by the Author:	22-Feb-2018
Complete List of Authors:	Zhao, Tian; University of Cambridge Medicine, Division of Cardiovascular Medicine<br>Kostapanos, Michalis; University of Cambridge Medicine, Experimental Medicine and Immunotherapeutics (EMIT) Division<br>Griffiths, Charmaine; Addenbrooke's Hospital, Clinical Trials Unit<br>Arbon, Emma; Addenbrooke's Hospital, Clinical Trials Unit<br>Hubsch, Annette; University of Cambridge Medicine, Experimental Medicine and Immunotherapeutics (EMIT) Division<br>Kaloyirou, Fotini; University of Cambridge Medicine, Experimental Medicine and Immunotherapeutics (EMIT) Division<br>Helmy, Joanna; University of Cambridge Medicine, Experimental Medicine and Immunotherapeutics (EMIT) Division<br>Hoole, Stephen; Papworth Hospital NHS Foundation Trust, Cardiology<br>Rudd, James; University of Cambridge Medicine, Division of Cardiovascular Medicine<br>Wood, Graham; Addenbrooke's Hospital<br>Burling, Keith; Cambridge University Hospitals NHS Foundation Trust, Clinical Biochemistry<br>Bond, Simon; Cambridge University Hospitals NHS Foundation Trust, Cambridge clinical trials unit<br>Cheriyan, Joseph; University of Cambridge Medicine, Department of Pharmacology<br>Mallat, Ziad; University of Cambridge Medicine, Division of Cardiovascular Medicine
Keywords:	Ischaemic heart disease < CARDIOLOGY, acute coronary syndrome, interleukin 2, regulatory T cells, Aldesleukin

SCHOLARONE™
Manuscripts

Low dose interleukin-2 in patients with stable ischaemic heart disease and acute coronary syndromes (LILACS): protocol and study rationale for a randomised, double-blind, placebo controlled, phase I/II clinical trial

Tian X. Zhao[1,2], Michalis Kostapanos[2], Charmaine Griffiths[3], Emma L. Arbon[3], Annette Hubsch[2], Fotini Kaloyirou[2], Joanna Helmy[2], Stephen P. Hoole[4], James H.F. Rudd[1], Graham Wood[5], Keith Burling[6], Simon Bond[3], Joseph Cheriyan[2,3], Ziad Mallat[1]

[1] Department of Medicine, Division of Cardiovascular Medicine, University of Cambridge, Cambridge, UK
[2] Experimental Medicine and Immunotherapeutics (EMIT) Division, Department of Medicine, University of Cambridge, Cambridge, UK
[3] Cambridge Clinical Trials Unit, Addenbrooke's Hospital, Cambridge, UK
[4] Department of Interventional Cardiology, Royal Papworth Hospital NHS Trust, Cambridge, UK
[5] Department of Immunology, Cambridge University Hospitals, Cambridge, UK
[6] Clinical Biochemistry, Cambridge University Hospitals, Cambridge, UK

Correspondence to: Tian Zhao (txz20@cam.ac.uk)

Word count: 6628

For peer review only

Abstract

Introduction:

Inflammation and dysregulated immune responses play a crucial role in atherosclerosis, underlying ischaemic heart disease (IHD) and acute coronary syndromes (ACS). Immune responses are also major determinants of the post-ischaemic injury in myocardial infarction. Regulatory T cells ($CD4^{+}CD25^{+}FOXP3^{+}$; Treg) induce immune tolerance and preserve immune homeostasis. Recent *in vivo* studies suggested that low-dose interleukin-2 (IL-2) can increase Treg cell numbers. Aldesleukin is a human recombinant form of IL-2 which has been used therapeutically in several autoimmune diseases. However, its safety and efficacy is unknown in the setting of coronary artery disease.

Method and analysis:

Low-dose interleukin-2 in patients with stable ischaemic heart disease and acute coronary syndromes (LILACS) is a single centre, first-in-class, dose escalation, two-part clinical trial. Patients with stable IHD (Part A) and ACS (Part B) will be randomised to receive either IL-2 (Aldesleukin; dose range 0.3 – 3 $x10^{6}$ IU) or placebo once daily, given subcutaneously, for 5 consecutive days. Part A will have 5 dose levels with 5 patients in each group. Group 1 will receive a dose of 0.3 $x10^{6}$ IU whilst the dose for the remaining 4 groups will be determined upon completion of the preceding group. Part B will have 4 dose levels with 8 patients in each group. The dose of the first group will be based on Part A. Doses for each of the subsequent 3 groups will similarly be determined after completion of the previous group. The primary endpoint is safety and tolerability of aldesleukin, and to determine the dose which increases mean circulating Treg levels by at least 75%.

Ethics and dissemination:

The study was given a favourable opinion by the Greater Manchester Central Research Ethics Committee, UK (REC:17/NW/0012). The results of this study will be reported, through peer-reviewed journals, conference presentations and an internal organisational report.

Trial registration numbers: Clinicaltrials.gov (NCT03113773)

Article summary

Strengths and limitations of this study

- The double-blind, placebo-controlled design will allow assessment of the safety and efficacy of low-dose IL-2 in patients with stable ischaemic heart disease and in acute coronary syndrome patients, both conditions where it is currently contraindicated
- The adaptive dose design of this study will allow assessment of the potential for low-dose IL-2 to increase mean circulating Treg levels by at least 75%
- Due to its early phase design, this study is not powered to assess any clinical outcome data for patients

Keywords: ischaemic heart disease, acute coronary syndrome, interleukin-2, regulatory T cells, Aldesleukin

For peer review only

Background

Despite major advances in the treatment, ischaemic heart disease (IHD) remains a significant cause of mortality and morbidity. It is now firmly established that inflammation and the immune response are crucial to the pathophysiology of IHD. This is true both in atherosclerosis which underlies stable angina and in progression to plaque instability and disruption in acute coronary syndrome (ACS) [1].

Although the innate immune system has been better studied in atherosclerosis, the role of the adaptive immune responses is now being increasingly understood. Several studies reported a perturbation of the T cell repertoire in ACS patients [2] with expansion of an effector and activated T cell subset [3], which is, at least in part, directed to antigens contained in the disrupted plaque [4]. Initial pre-clinical findings have shown that regulatory T (Treg) cell mediated immunity reduces experimental atherosclerosis and plaque inflammation [5].

In ACS patients, there is an imbalance between T effector and Treg cells. Despite the effector T cell compartment activation, the percentage and function of circulating Tregs appear to be significantly decreased in in the setting of ACS [6]-[9]. This imbalance is thought to play a key role in coronary plaque progression and destabilisation. In this context, low levels of circulating baseline $CD4^+Foxp3^+$ Treg cells were associated with an increased risk for acute coronary events in the Malmö Diet and Cancer Study [10].

Following myocardial infarction, the ischaemic and necrotic myocardial tissue may present self-antigens to the immune system, leading to antigen-specific, autoimmune adaptive responses [11], [12]. Recent studies indicate that $CD4^+$ T cells, and particularly Treg cells, are important for the control of post-ischaemic immune responses and the promotion of myocardial healing [12]-[15]. In another study, inhibition of Treg recruitment to the site of myocardial injury resulted in excessive post-ischaemic inflammation, matrix degradation and adverse remodelling [13]. In contrast, *in vivo* expansion of Treg cells or their therapeutic activation by superagonistic anti-CD28 antibodies attenuated left ventricular remodelling and improved cardiac function [14], [15].

Interleukin-2 (IL-2) plays a key role in Treg cell development, expansion, survival and suppressive function [16], [17]. Deficiency of either IL-2 or IL-2 receptor in mice greatly compromises Treg development and promotes autoimmune responses [18]. Supplementation of IL-2 substantially increases Treg cell levels and significantly limits plaque development and inflammation in mice prone to atherosclerosis [19], [20]. Treg cells show a much lower threshold response to IL-2 receptor signalling compared to effector T cells. This led to the hypothesis that, in contrast to high dose IL-2 designed to activate T effector cells in cancer, supplementation with low doses of IL-2 in the setting of T cell-mediated immune diseases could selectively promote the expansion of Treg cells at the expense of T effector cells, thereby limiting harmful immune responses. This hypothesis was initially confirmed in two pilot human clinical studies in two different disease settings, graft-versus-host disease [21], [22] and in hepatitis C virus-induced vasculitis [23]. In both studies, administration of low doses of IL-2 in the form of Aldesleukin (daily administration of 0.3×10^6 to 3.0×10^6 IU IL-2 per square meter of body-surface area for 8 weeks, or repetitive

For peer review only - http://bmjopen.bmj.com/site/about/guidelines.xhtml
For peer review only

5-day courses of 1.0 x10^6 to 3.0 x10^6 IU IL-2) led to a rapid and marked expansion of the circulating pool of Treg cells, which were at least doubled without affecting the pool of conventional CD4$^+$ T (i.e. T effector) cells. The expanded Tregs retained potent suppressive functions and the treatment was associated with a reduction in the inflammatory response and a concomitant clinical improvement in a substantial proportion of patients. Treatment with low dose IL-2 was safe and no adverse effects were reported. This strategy is currently being adapted and tested in various disease settings, where Treg cell promotion is believed to be of potential therapeutic benefit [22]-[25]. In this trial, we hypothesise that low dose IL-2 (Aldesleukin) can be used in IHD to increase Treg numbers and to rebalance the immune system with the overall goal of decreasing recurrent myocardial infarction and cardiovascular death.

Aldesleukin (Proleukin®, Novartis) is a commercially available (Marketing Authorisation Holder: Novartis Pharmaceuticals UK Limited) IL-2 licensed for the treatment of metastatic renal cell carcinoma in the UK. It is produced by recombinant DNA technology using an Escherichia coli strain, which contains a genetically engineered modification of the human IL-2 gene, and is administered either intravenously (IV) or subcutaneously (SC). Following short IV infusion, its pharmacokinetic profile is typified by high plasma concentrations, rapid distribution into the extravascular space and a rapid renal clearance. The recommended doses for continuous infusion and subcutaneous injection (as detailed in the SmPC) are repeated cycles of 18 x 10^6 IU per m^2 per 24-hours for 5 days, and repeated doses of 18 x 10^6 IU respectively. Peak plasma levels are reached in 2-6 hours after subcutaneous administration, with bioavailability of Aldesleukin ranging between 31–47%. The process of absorption and elimination of subcutaneous Aldesleukin is described by a one-compartment model, with a 45-minute absorption half-life and a 3-5 hour elimination half-life [26].

Use of IL-2 in clinical trials to date

The first report of effective IL-2 therapy in human cancer trials was published in 1985 [27]. The trial patients in that study were placed on dose-escalated IL-2 regimens, of up to approximately 120 Million IU (MIU). Associated with these high IL-2 doses were side effects such as capillary leak syndrome (which is characterized by a loss of vascular tone and extravasation of plasma proteins and fluid into the extravascular space, ultimately resulting in hypotension, tachycardia, dyspnoea and pulmonary oedema), and kidney and liver damage (both characterised by increased serum creatinine and bilirubin levels respectively).

The use of low dose IL-2 to expand Treg cell populations in autoimmune and allo-inflammatory conditions has been previously explored and published in human clinical trials. In these studies, patients received at least 1 dose of IL-2 ranging from 0.3x10^6 IU – 3.0x10^6 IU. In two studies of 12 and 21 healthy volunteers respectively, there were minimal adverse events (AEs), consisting mainly of grade 1 injection site reactions. No cardiovascular AEs were noted [28], [29]. In one phase 1/2a study, 24 patients with diabetes mellitus were recruited and given a maximum dose of 3.0x10^6 IU daily for 5 days. The authors found that IL-2 was well tolerated at all doses, with no serious adverse events (SAEs). However, there was a dose-response relationship for non-serious AEs. The most common AEs in the treatment phase were injection-site reactions and an influenza-like syndrome [30]. In a later trial of 40 type 1 diabetics, the authors found that doses of Aldesleukin were well tolerated

For peer review only - http://bmjopen.bmj.com/site/about/guidelines.xhtml

at all doses, with no serious adverse events (SAEs) reported. The majority of participants had an expected AE at the injection site consisting a non-itchy, local (1–5 cm), non-painful erythematous rash which resolved on average by day 10 [31]. No cardiovascular AEs were reported in either study. Low dose IL-2 has also been used in 38 SLE patients[25], who are considered to have a higher risk of coronary artery disease and therefore cardiac events [32]. However, no SAEs were observed whilst injection site reactions and flu like symptoms were observed in 13.2% and 5.3% of patient respectively [25].

Nevertheless, IL-2 is contraindicated in patients with a significant history, or current evidence of, severe cardiac disease. Therefore, we sought to determine the safety and efficacy of low dose Aldesleukin in patients with pre-existing cardiac conditions. We hypothesise that low dose IL-2, unlike higher doses, can be safely administered and is effective in expanding the Treg population in patients with stable and acute coronary artery disease. In this trial, low dose IL-2 will initially be administered in stable IHD patients at escalating doses and, following safety reviews, will be given to ACS patients. The Treg response data from the ACS population will help select the most appropriate dose to assess efficacy in clinical trials in ACS.

Method

This is a prospective single centre, randomised, double-blind, placebo controlled, phase 1/2 clinical trial. It will be performed at the National Institute for Health Research/Wellcome Trust Cambridge Clinical Research Centre, Cambridge University Hospitals, Cambridge, UK with overall study co-ordination provided by the Cambridge Clinical Trials Unit, Cambridge University Hospitals NHS Foundation Trust.

Study populations

The trial will be performed in two parts. Part A will include patients with stable IHD aged 18-75 years with a clinical diagnosis of IHD for more than 6 months. The inclusion and exclusion criteria are detailed in Table 1, but in summary, patients with a myocardial infarction within the last 6 months, cardiogenic shock, hypo/hypertension, heart failure (EF<45%), pro-arrhythmogenic conditions, renal, hepatic, thyroid or haematological dysfunction, active infection, poorly controlled diabetes, active autoimmune disease, current malignancy, history of seizures or immunosuppression will be excluded from this part of the study.

Part B will be performed after Part A and will include patients aged of 18-85 years admitted with a diagnosis of non-ST elevation myocardial infarction. The inclusion and exclusion criteria are detailed in Table 1. In brief, patients with cardiogenic shock, hyper/hypotension, heart failure (ejection fraction<35%), long QT or arrhythmias, renal, hepatic, thyroid or haematological dysfunction, active infection, active autoimmune disease, current malignancy, history of seizures or immunosuppression will be excluded from this part of the study.

Study protocol

For peer review only

Part A

Patients will be recruited from advertisements, outpatient clinics or research databases. Participants will have at least 24 hours to review the Patient Information Sheet prior to informed consent. Study procedures will only be conducted following formal written consent at the screening visit 1 (V1). Baseline blood tests will consist of electrolytes, renal, liver and thyroid function, full blood count, clotting profile, Hepatitis B/C and HIV screening, HbA1c, and pregnancy screen (where applicable). Baseline vitals, electrocardiogram (ECG), echocardiogram, clinical history and physical examination will also be performed. Randomisation will be carried out via a paper based concealment list generated by a statistician.

The trial design is described in detail in Figure 1. In brief, following randomisation at V1, patients will attend 5 consecutive daily outpatient visits (V2-6) during which blinded subcutaneous injections of Aldesleukin or placebo will be administered. At each visit, prior to the study drug administration, and the medical history will be obtained/reviewed, along with a physical examination, baseline vitals, safety bloods and a 12 lead-ECG. Patients will have continuous cardiac monitoring during each visit for at least 30minutes pre- and 1.5 hours post-dose. After dosing, a series of ECGs will be performed at 15 minutes, 30 minutes and 60 minutes whilst vitals will be assessed at 30 minutes and 60 minutes. For each dosing visit, serum IL-2 levels will be taken at baseline and at 90 minutes post-dose.

There are 2 follow up visits (V7 and V8). Assessments during both visits will include a medical review, physical examination, vitals, ECGs and safety bloods tests. Additionally, at V8, a follow up thyroid function blood test and echocardiogram will be performed.

In addition, during visits V2, V7 (Figure 1), cardiac biomarkers (high sensitivity C-reactive protein (hs-CRP), IL-6, brain-type natriuretic peptide (BNP) and troponin) and lymphocyte subsets (including Treg level, see below) analysis will be performed. During visit V8, cardiac biomarkers will be re-assessed.

A total of 25 patients, 5 in each of the 5 dosing groups, will be included in Part A (drug:placebo ratio of 3:2). In line with current Phase 1 designs, a sentinel dosing approach will be employed whereby the first 2 patients of each group will be allocated to either Aldesleukin or placebo in a random order. After a safety review of these first 2 patients, the remaining patients will then be dosed (see Figure 2).

Part B

Patients with a primary diagnosis of non-ST elevation myocardial infarction will be recruited from the medical and cardiology wards at Cambridge University Hospitals NHS Foundation Trust.. Participants will be given at least 24 hours to review the Patient Information Sheet prior to formal consent. Dosing should commence within 8 days of screening. All visits and blinding procedures will be the same as Part A. However, in part B, a total of 32 patients will be recruited, 8 patients in each of the 4 dosing groups (drug:placebo ratio of 6:2). A sentinel approach to dosing will also be employed in each group. After a safety review of the first 2 patients, the remaining cohort will be randomly allocated to study treatments as shown in Figure 3. The visit schedule for each patient is the same as Part A (Figure 1).

For peer review only - http://bmjopen.bmj.com/site/about/guidelines.xhtml

Dose escalation strategy

The first group of patients in Part A will receive $0.3x10^6$ IU of Aldesleukin daily. Thereafter, a blinded review of patient data by the blinded Trial Management Group (TMG) including review of adverse events, blood results, ECGs, clinical records and where possible, drug pharmacodynamics and pharmacokinetics. The TMG will comprise of a lead physician (LILACS Chief Investigator), a research physician/scientist, a research nurse, trial co-ordinator and an unblinded study statistician (for the purposes of data analysis). All data presented to the TMG will be in a blinded manner. The dose in the second group will be determined after this review and the same process will be followed in each of the following groups. The maximum dose increments allowed by the protocol between groups will be double the previous dose, and capped to a maximum $3.0\ x10^6$ IU.

Following completion of Part A, an unblinded independent Data Monitoring Committee (DMC) will review all available safety data. The DMC will be comprised of an independent group of physicians who will determine whether it is safe to progress to Part B, based on available safety and pharmacodynamic data. After this analysis, the dose in each group will be determined based on the review of ongoing patient data by the Trial Management Group, as in Part A previously. The protocol mandates that the maximum dose used in Part B will not exceed that of Part A.

Outcome measure

Part A

The primary outcome will be the safety of IL-2 in patients. This will be assessed through:

- A review of AEs and SAEs, and concomitant medications
- Changes in safety bloods (electrolytes, bone profile, serum creatinine, liver function tests, thyroid function tests, blood glucose, full blood count and differential, clotting)
- 12-Lead ECG and cardiac monitoring changes (arrhythmias, ischaemic changes, QTcB)
- Vital observations
- Echocardiogram changes at baseline and follow up

Exploratory endpoints will include:

- Change in the mean circulating Treg level measured by fluorescence activated cell sorting (FACS) analysis following treatment with IL-2, over the 5 days of the treatment period.
- Cardiac biomarker measurements including hs-CRP, troponin I, IL-6 and b-type natriuretic peptide) from analysed blood samples.
- Change in lymphocyte subsets measured by FACS analysis
- Pharmacokinetic analysis of IL-2 levels

For peer review only - http://bmjopen.bmj.com/site/about/guidelines.xhtml

Part B

As with Part A, the primary endpoint will be safety and tolerability of IL-2. A further primary endpoint will be the change in mean circulating Treg levels and whether IL-2 increases mean circulating Treg levels by at least 75% over the 5 days of the treatment period. Exploratory endpoints are the same as for Part A.

Adverse event reporting

The International Conference on Harmonisation definitions are used for adverse events (AEs), adverse reactions (ARs), severe AE/ARs (SAEs/ SARs) and suspected unexpected SARs (SUSARs). A medically qualified doctor will determine the relationship of each AE to the study drug as either 'related' (reasonable temporal sequence and not reasonably attributed to another cause) or 'not related'. They will also make an assessment on the seriousness of the event. Changes in laboratory values are only considered to be AEs if they are judged to be clinically significant or if intervention is required. It is left to the investigator's clinical judgment whether an AE is of sufficient severity to require the volunteer's removal from the trial.

Lymphocyte analysis

Lymphocyte subset analysis will be performed at the Department of Clinical Immunology, Cambridge University Hospitals, Cambridge, UK, within 4 hours of sample collection in EDTA. Laboratory technicians will be blinded to treatment allocation. The antibodies that will be used are anti-CD3 (clone SK7, phycoerythrin [PE]-Cy7-labelled; BD Biosciences), anti-CD4 (clone RPA-T4, FITC-labelled; BD Biosciences), anti-CD127 (clone HIL-7R-M21, PE-labelled; BD Biosciences), anti-CD25 (clone M-A251 and 2A3, allophycocyanin [APC]-labelled; BD Biosciences), anti-CD45RA (clone HI100, APC-Cy7-labelled; BioLegend), and anti-CD62L (clone DREG-56, PerCP/Cy5.5-labelled; BioLegend). Whole blood will be analysed by performing clinical FACS assays to measure absolute lymphocyte counts, proportions of lymphocytes, and CD25 expression on Treg cells. Defined concentrations of fluorescently labelled beads are added to whole blood and analysed to accurately count the absolute number of lymphocytes, $CD3^+$ T cells, $CD4^+$ and $CD8^+$ T cells, $CD19^+$ B cells and $CD19^-$ $CD16^+$ $CD56^+$ NK cells as a percentage of all lymphocytes. In a parallel whole blood FACS assay, a lymphocyte gate is drawn to include all events. The $CD3^+$, $CD4^+$ T-cell gate excludes $CD8^+$ T cells and B cells. Tregs are defined by $CD3^+CD4^+CD25^{high}CD127^{low}$ makers. A cocktail of six standardised beads labelled with different amounts of fluorescent allophycocyanin (APC) are measured by FACS daily to accurately measure CD25-APC on the surface of Tregs and a standard curve plotted. The mean fluorescence intensity of $CD25^+$ on Tregs can be accurately read from the curve, minimising interassay variation. $CD127^{low}$, $CD25^+$ T regulatory cells (Tregs) are separated from non-Tregs and this percentage is used to calculate the absolute Treg count out of $CD3^+CD4^+$ T cells. Among the T effector (non-Treg) $CD43^+CD4^+$ population, we define naïve effectors $CD45RA^+CD62L^+$, effector memory $CD45RA^-CD62L^-$, central memory $CD45RA^-CD62L^+$, and effector memory CD45 RA^+(TEMRA) $CD45RA^+CD62L^-$ cells. Total memory effectors are the sum of central memory and effector memory cells.

For peer review only - http://bmjopen.bmj.com/site/about/guidelines.xhtml

Cardiac biomarkers

Blood will be taken in gel serum tubes and the serum will be banked and analysed at the Core Biochemical Assay Laboratory, Cambridge. Hs-CRP and NT-proBNP will be measured by immunoassays on the Siemens Dimension EXL autoanalyser. All reagents and calibrators are supplied by Siemens and assays will be performed according to the manufacturer's instructions.

IL-6 will be measured in duplicate using ultra-sensitive electrochemical luminescence immunoassay on the Mesoscale Discovery assay platform and read on the MesoScale Diagnostics Sector Imager 6000. All reagents and calibrators will be supplied by MesoScale Discovery.

Stopping criteria

Dose escalation stopping criteria will be met if 2 patients within a trial group experience any combination of: a SAE defined as possibly, probably or definitely related to the trial drug (i.e. it is a SAR), an adverse event that is severe and at least possibly related to the trial drug, or any of the objective stopping criteria detailed Table 2. The following will then occur:

- Dosing will be immediately discontinued for the patients experiencing the event
- Dosing will be halted for all other patients currently in the treatment period of the trial (i.e. patients receiving treatment in the same group)
- A safety review by the independent Data Monitoring Committee (DMC) will be conducted to determine how to proceed with the trial
- Any further single instances of the events outlined above for the same group will trigger a further DMC safety review
- Any patients who have their dosing discontinued will be withdrawn from the trial

Statistical methods and data handling

This is an exploratory study that is not designed to formally test a hypothesis in a confirmatory fashion. Given that both parts of the trial have clinical safety as primary endpoints, a formal power calculation is not relevant. A sample size of 57 patients is achievable within the proposed time scale, given the size of the targeted patient population at our study site. The frequency of adverse events per patient will be summarised for each event based on dose level. Summary statistics of laboratory values by dose and visit will be produced where required. The statistician will use the data from each group to perform a modelling analysis of the effect of Aldesleukin based on dose and effect size using a smoothed line plot of the mean and 95% confidence internals. Generally summary statistics of continuous variables will report mean, median, SD, min and max, although a log-transformed scale may be used where the data are skewed. Binary or categorical variables will be summarised using the p% (x/n) format. The Treg data and other secondary biomarker endpoints will be summarised with individual patient profiles over time, and

For peer review only - http://bmjopen.bmj.com/site/about/guidelines.xhtml

summary statistics broken down by dose and visit. Formal estimates of the differences between doses will be made at each time point with accompanying 95% confidence intervals and p-values.

Subjects will be coded by a numeric code to create an anonymous dataset. All data will be transferred into a Case Report Form, which will be coded onto a MACRO database.

Patient and public involvement

Heart attacks are distressing and impacts patients' lives dramatically [33]. The aim of this research is to help ameliorate this issue and potentially reduce reoccurrence. Lay members of the ethics committee reviewed this study and made constructive comments which have been addressed. Patients were not involved in the recruitment to or conduct of the study. All patients provided full informed consent, with at least 24 hours to consider the information and at least two opportunities to discuss the trial in detail with the investigators. The results of the study will be disseminated to all study patients at the end of the trial.

Study timeline

The trial began on the 15th of May 2017. The anticipated final follow-up visit(s) will be in January 2019. Primary analyses are projected to be completed by February 2019.

Ethics and dissemination

The study was given a favourable opinion by the Greater Manchester Central Research Ethics Committee, UK (REC: 17/NW/0012), and approved by the UK's Health Research Authority. The MHRA formally granted regulatory acceptance on 28th April 2017. All study procedures will be conducted after formal written consent, in accordance with the Declaration of Helsinki. The trial is registered on clinicaltrials.gov (NCT03113773) prior to trial commencement, and the results of this study will be published in a peer reviewed journal after completion.

Funding statement and acknowledgements

This work was funded by the Medical Research Council, grant number MR/N028015/1 and the British Heart Foundation Cambridge Centre of Excellence (RCAG/521). JC acknowledges funding support from the Cambridge Comprehensive Biomedical Research Centre. ZM acknowledges funding support from the British Heart Foundation for his BHF Chair. JHFR acknowledges funding support from the HEFCE, the NIHR Cambridge Biomedical Research Centre, the British Heart Foundation, the EPSRC and the Wellcome Trust. We acknowledge support from the Cambridge Clinical Research Centre and the Core Biochemistry Assay Laboratory in the conduct of this trial and its endpoints.

Authors' contributions and competing interests

For peer review only

TZ, MK, SPH, JC and ZM contributed to the writing of this manuscript. TZ, MK, CG, EA, AH, FK, JH, JHFR, GW, KB, SB, JC and ZM contributed to the writing of the protocol. The authors do not report any competing interests.

References

[1] P. Libby, P. M. Ridker, G. K. Hansson, Leducq Transatlantic Network on Atherothrombosis, “Inflammation in atherosclerosis: from pathophysiology to practice.,” *Journal of the American College of Cardiology*, vol. 54, no. 23, pp. 2129–2138, Dec. 2009.

[2] G. Liuzzo, L. M. Biasucci, G. Trotta, S. Brugaletta, M. Pinnelli, G. Digianuario, V. Rizzello, A. G. Rebuzzi, C. Rumi, A. Maseri, and F. Crea, “Unusual CD4+CD28nullT Lymphocytes and Recurrence of Acute Coronary Events,” *Journal of the American College of Cardiology*, vol. 50, no. 15, pp. 1450–1458, Oct. 2007.

[3] E. Ammirati, A. C. Vermi, D. Cianflone, M. Banfi, C. Foglieni, C. Godino, F. Airoldi, L. A. Ferri, C. L. Gorman, A. A. Manfredi, A. Maseri, A. P. Cope, and C. Monaco, “Expansion of T-Cell Receptor dim Effector T Cells in Acute Coronary Syndromes,” *Arteriosclerosis, Thrombosis, and Vascular Biology*, vol. 28, no. 12, pp. 2305–2311, Nov. 2008.

[4] G. Caligiuri, G. Paulsson, A. Nicoletti, A. Maseri, and G. K. Hansson, “Evidence for antigen-driven T-cell response in unstable angina.,” *Circulation*, vol. 102, no. 10, pp. 1114–1119, Sep. 2000.

[5] H. Ait-Oufella, B. L. Salomon, S. Potteaux, A.-K. L. Robertson, P. Gourdy, J. Zoll, R. Merval, B. Esposito, J. L. Cohen, S. Fisson, R. A. Flavell, G. K. Hansson, D. Klatzmann, A. Tedgui, and Z. Mallat, “Natural regulatory T cells control the development of atherosclerosis in mice,” *Nature Medicine*, vol. 12, no. 2, pp. 178–180, Feb. 2006.

[6] A. Mor, G. Luboshits, D. Planer, G. Keren, and J. George, “Altered status of CD4+CD25+ regulatory T cells in patients with acute coronary syndromes,” *European Heart Journal*, vol. 27, no. 21, pp. 2530–2537, Sep. 2006.

[7] S.-F. Han, P. Liu, W. Zhang, L. Bu, M. Shen, H. Li, Y.-H. Fan, K. Cheng, H.-X. Cheng, C.-X. Li, and G.-L. Jia, “The opposite-direction modulation of CD4+CD25+ Tregs and T helper 1 cells in acute coronary syndromes,” *Clinical Immunology*, vol. 124, no. 1, pp. 90–97, Jul. 2007.

[8] X. Cheng, X. Yu, Y.-J. Ding, Q.-Q. Fu, J.-J. Xie, T.-T. Tang, R. Yao, Y. Chen, and Y.-H. Liao, “The Th17/Treg imbalance in patients with acute coronary syndrome,” *Clinical Immunology*, vol. 127, no. 1, pp. 89–97, Apr. 2008.

[9] E. Ammirati, D. Cianflone, M. Banfi, V. Vecchio, A. Palini, M. De Metrio, G. Marenzi, C. Panciroli, G. Tumminello, A. Anzuini, A. Palloshi, L. Grigore, K. Garlaschelli, S. Tramontana, D. Tavano, F. Airoldi, A. A. Manfredi, A. L. Catapano, and G. D. Norata, “Circulating CD4+CD25hiCD127lo Regulatory T-Cell Levels Do Not Reflect the Extent or Severity of Carotid and Coronary Atherosclerosis,” *Arteriosclerosis, Thrombosis, and Vascular Biology*, vol. 30, no. 9, pp. 1832–1841, Aug. 2010.

[10] M. Wigren, H. Björkbacka, L. Andersson, I. Ljungcrantz, G. N. Fredrikson, M. Persson, C. Bryngelsson, B. Hedblad, and J. Nilsson, “Low levels of circulating CD4+FoxP3+ T cells are associated with an increased risk for development of myocardial infarction but not for stroke.,” *Arteriosclerosis, Thrombosis, and Vascular Biology*, vol. 32, no. 8, pp. 2000–2004, Aug. 2012.

[11] M. Zhang, E. M. Alicot, I. Chiu, J. Li, N. Verna, T. Vorup-Jensen, B. Kessler, M. Shimaoka, R. Chan, D. Friend, U. Mahmood, R. Weissleder, F. D. Moore, and M. C. Carroll, “Identification of the target self-antigens in reperfusion injury.,” *J. Exp. Med.*, vol. 203, no. 1, pp. 141–152, Jan. 2006.

[12] U. Hofmann, N. Beyersdorf, J. Weirather, A. Podolskaya, J. Bauersachs, G. Ertl, T. Kerkau, and S. Frantz, “Activation of CD4+ T lymphocytes improves wound healing and survival after experimental myocardial infarction in mice.,” *Circulation*, vol. 125, no. 13, pp. 1652–1663, Apr. 2012.

[13] M. Dobaczewski, Y. Xia, M. Bujak, C. Gonzalez-Quesada, and N. G. Frangogiannis, “CCR5 Signaling Suppresses Inflammation and Reduces Adverse Remodeling of the Infarcted Heart, Mediating Recruitment of Regulatory T Cells,” *The American Journal of Pathology*, vol. 176, no. 5, pp. 2177–2187, May 2010.

[14] K. Matsumoto, M. Ogawa, J.-I. Suzuki, Y. Hirata, R. Nagai, and M. Isobe, “Regulatory T

For peer review only - http://bmjopen.bmj.com/site/about/guidelines.xhtml

lymphocytes attenuate myocardial infarction-induced ventricular remodeling in mice.," *Int Heart J*, vol. 52, no. 6, pp. 382–387, 2011.

[15] T.-T. Tang, J. Yuan, Z.-F. Zhu, W.-C. Zhang, H. Xiao, N. Xia, X.-X. Yan, S.-F. Nie, J. Liu, S.-F. Zhou, J.-J. Li, R. Yao, M.-Y. Liao, X. Tu, Y.-H. Liao, and X. Cheng, "Regulatory T cells ameliorate cardiac remodeling after myocardial infarction.," *Basic Res. Cardiol.*, vol. 107, no. 1, p. 232, Jan. 2012.

[16] T. R. Malek and A. L. Bayer, "Tolerance, not immunity, crucially depends on IL-2.," *Nat. Rev. Immunol.*, vol. 4, no. 9, pp. 665–674, Sep. 2004.

[17] K. Wing and S. Sakaguchi, "Regulatory T cells exert checks and balances on self tolerance and autoimmunity.," *Nat. Immunol.*, vol. 11, no. 1, pp. 7–13, Jan. 2010.

[18] T. R. Malek, "The biology of interleukin-2.," *Annu. Rev. Immunol.*, vol. 26, no. 1, pp. 453–479, 2008.

[19] A. C. Foks, V. Frodermann, M. ter Borg, K. L. L. Habets, I. Bot, Y. Zhao, M. van Eck, T. J. C. van Berkel, J. Kuiper, and G. H. M. van Puijvelde, "Differential effects of regulatory T cells on the initiation and regression of atherosclerosis.," *Atherosclerosis*, vol. 218, no. 1, pp. 53–60, Sep. 2011.

[20] T. N. Dinh, T. S. Kyaw, P. Kanellakis, K. To, P. Tipping, B.-H. Toh, A. Bobik, and A. Agrotis, "Cytokine therapy with interleukin-2/anti-interleukin-2 monoclonal antibody complexes expands CD4+CD25+Foxp3+ regulatory T cells and attenuates development and progression of atherosclerosis.," *Circulation*, vol. 126, no. 10, pp. 1256–1266, Sep. 2012.

[21] J. Koreth, K.-I. Matsuoka, H. T. Kim, S. M. McDonough, B. Bindra, E. P. Alyea, P. Armand, C. Cutler, V. T. Ho, N. S. Treister, D. C. Bienfang, S. Prasad, D. Tzachanis, R. M. Joyce, D. E. Avigan, J. H. Antin, J. Ritz, and R. J. Soiffer, "Interleukin-2 and regulatory T cells in graft-versus-host disease.," *N Engl J Med*, vol. 365, no. 22, pp. 2055–2066, Dec. 2011.

[22] K.-I. Matsuoka, J. Koreth, H. T. Kim, G. Bascug, S. McDonough, Y. Kawano, K. Murase, C. Cutler, V. T. Ho, E. P. Alyea, P. Armand, B. R. Blazar, J. H. Antin, R. J. Soiffer, and J. Ritz, "Low-dose interleukin-2 therapy restores regulatory T cell homeostasis in patients with chronic graft-versus-host disease.," *Sci Transl Med*, vol. 5, no. 179, pp. 179ra43–179ra43, Apr. 2013.

[23] D. Saadoun, M. Rosenzwajg, F. Joly, A. Six, F. Carrat, V. Thibault, D. Sene, P. Cacoub, and D. Klatzmann, "Regulatory T-cell responses to low-dose interleukin-2 in HCV-induced vasculitis.," *N Engl J Med*, vol. 365, no. 22, pp. 2067–2077, Dec. 2011.

[24] M. Rosenzwajg, G. Churlaud, R. Mallone, A. Six, N. Dérian, W. Chaara, R. Lorenzon, S. A. Long, J. H. Buckner, G. Afonso, H.-P. Pham, A. Hartemann, A. Yu, A. Pugliese, T. R. Malek, and D. Klatzmann, "Low-dose interleukin-2 fosters a dose-dependent regulatory T cell tuned milieu in T1D patients.," *J. Autoimmun.*, vol. 58, pp. 48–58, Apr. 2015.

[25] J. He, X. Zhang, Y. Wei, X. Sun, Y. Chen, J. Deng, Y. Jin, Y. Gan, X. Hu, R. Jia, C. Xu, Z. Hou, Y. A. Leong, L. Zhu, J. Feng, Y. An, Y. Jia, C. Li, X. Liu, H. Ye, L. Ren, R. Li, H. Yao, Y. Li, S. Chen, X. Zhang, Y. Su, J. Guo, N. Shen, E. F. Morand, D. Yu, and Z. Li, "Low-dose interleukin-2 treatment selectively modulates CD4+ T cell subsets in patients with systemic lupus erythematosus," *Nature Medicine*, vol. 22, no. 9, pp. 991–993, Aug. 2016.

[26] Novartis Pharmaceuticals UK Ltd, "Proleukin Summary of Product Characteristics Updated 20-Jan-2015."

[27] S. A. Rosenberg, M. T. Lotze, L. M. Muul, S. Leitman, A. E. Chang, S. E. Ettinghausen, Y. L. Matory, J. M. Skibber, E. Shiloni, and J. T. Vetto, "Observations on the systemic administration of autologous lymphokine-activated killer cells and recombinant interleukin-2 to patients with metastatic cancer.," *N Engl J Med*, vol. 313, no. 23, pp. 1485–1492, Dec. 1985.

[28] S. Ito, M. Battiwalla, J. J. Melenhorst, M. Carlsten, A. Biancotto, E. Wang, Z. Xie, P. Muranski, M. J. Olnes, K. Tanimoto, P. Schum, S. Perl, H. Dickler, J. P. McCoy, T. Hughes, L. Bojanowski, N. F. Hensel, N. S. Young, and A. J. Barrett, "Ultra-Low Dose IL-2 Safely Expands Regulatory T Cells and CD56 NK Cells in Healthy Volunteers: Towards Safer Stem Cell Donors?," *Blood*, vol. 120, no. 21, p. 3283, Oct. 2015.

[29] S. Ito, C. M. Bollard, M. Carlsten, J. J. Melenhorst, A. Biancotto, E. Wang, J. Chen, Y. Kotliarov, F. Cheung, Z. Xie, F. Marincola, K. Tanimoto, M. Battiwalla, M. J. Olnes, S. Perl, P. Schum, T. E. Hughes, K. Keyvanfar, N. Hensel, P. Muranski, N. S. Young, and A. J. Barrett, "Ultra-low dose interleukin-2 promotes immune-modulating function of regulatory T cells and natural killer cells in healthy volunteers.," *Mol. Ther.*, vol. 22, no. 7, pp. 1388–1395, Jul. 2014.

[30] A. Hartemann, G. Bensimon, C. A. Payan, S. Jacqueminet, O. Bourron, N. Nicolas, M.

For peer review only

Fonfrede, M. Rosenzwajg, C. Bernard, and D. Klatzmann, “Low-dose interleukin 2 in patients with type 1 diabetes: a phase 1/2 randomised, double-blind, placebo-controlled trial.,” *Lancet Diabetes Endocrinol*, vol. 1, no. 4, pp. 295–305, Dec. 2013.
[31] J. A. Todd, M. Evangelou, A. J. Cutler, M. L. Pekalski, N. M. Walker, H. E. Stevens, L. Porter, D. J. Smyth, D. B. Rainbow, R. C. Ferreira, L. Esposito, K. M. D. Hunter, K. Loudon, K. Irons, J. H. Yang, C. J. M. Bell, H. Schuilenburg, J. Heywood, B. Challis, S. Neupane, P. Clarke, G. Coleman, S. Dawson, D. Goymer, K. Anselmiova, J. Kennet, J. Brown, S. L. Caddy, J. Lu, J. Greatorex, I. Goodfellow, C. Wallace, T. I. Tree, M. Evans, A. P. Mander, S. Bond, L. S. Wicker, and F. Waldron-Lynch, “Regulatory T Cell Responses in Participants with Type 1 Diabetes after a Single Dose of Interleukin-2: A Non-Randomised, Open Label, Adaptive Dose-Finding Trial.,” *PLoS Med.*, vol. 13, no. 10, p. e1002139, Oct. 2016.
[32] C. M. Bartels, K. A. Buhr, J. W. Goldberg, C. L. Bell, M. Visekruna, S. Nekkanti, and R. T. Greenlee, “Mortality and cardiovascular burden of systemic lupus erythematosus in a US population-based cohort.,” *J. Rheumatol.*, vol. 41, no. 4, pp. 680–687, Apr. 2014.
[33] S. L. Grace, S. E. Abbey, J. Irvine, Z. M. Shnek, and D. E. Stewart, “Prospective examination of anxiety persistence and its relationship to cardiac symptoms and recurrent cardiac events.,” *Psychother Psychosom*, vol. 73, no. 6, pp. 344–352, Nov. 2004.

For peer review only

For peer review only - http://bmjopen.bmj.com/site/about/guidelines.xhtml

Tables and figure legends

Table 1. Trial inclusion and exclusion criteria for parts A and B

Part A	Inclusion criteria	• Age 18-75 years old inclusive<br>• Previous history (> 6 months) of coronary artery disease<br>• No history of recent (< 6 months) admissions for an unstable cardiovascular event e.g. MI, unstable angina, ACS<br>• Written informed consent for participation in the trial
	Exclusion criteria	• Current presentation with cardiogenic shock, severe congestive heart failure and/or pulmonary oedema<br>• Known active bleeding or bleeding diatheses<br>• Known active infection requiring antibiotic treatment<br>• Severe hematologic abnormalities (haematocrit <30% and platelet cell count of <100 × 10^3/µL and white blood cell count <4 × 10^3/µL)<br>• Known malignancies requiring active treatment or follow up<br>• Known heart failure (LV EF <45%)<br>• Hypotension (Systolic BP<100mm Hg, DBP<50mmHg) at screening<br>• Uncontrolled hypertension (>160/100 mmHg) at screening<br>• History of recurrent syncope suggestive of arrhythmia syncope<br>• Known hepatic failure or abnormal LFTs at baseline (ALT > 2 x ULN, TBL > 1.5 x ULN and ALP > 1.5 x ULN)<br>• Acute kidney injury or chronic kidney disease at Stage > 3B (eGFR < 45 ml/min/1.73m^2)<br>• Known hyper- or hypothyroidism<br>• History of drug induced Stevens Johnson syndrome, Drug reaction with eosinophilia and systemic symptoms (DRESS syndrome) or toxic epidermal necrolysis<br>• History of recurrent epileptic seizures in the previous 4 years<br>• If known diabetic, uncontrolled diabetes defined as HbA1c > 64 mmol/mol<br>• Average corrected QT interval > 450 msecs<br>• Known chronic active hepatitis (B or C) or HIV infection<br>• Known autoimmune disease requiring active immunosuppressive therapy<br>• History of organ transplantation<br>• Any oral or intravenous Immunosuppressive treatment<br>• Known pregnancy or on-going lactation<br>• Current participation in other interventional clinical trials<br>• Contraindication or hypersensitivity to IL-2 treatment
Part B	Inclusion criteria	• Age 18-85 years old inclusive<br>• Current admission (on at least screening visit) with NSTEMI<br>• Willingness to be dosed within 8 days from initial date of current admission for ACS

For peer review only

For peer review only - http://bmjopen.bmj.com/site/about/guidelines.xhtml

		• Written informed consent for participation in the trial
	Exclusion criteria	• Current presentation with cardiogenic shock, electrical instability, severe congestive heart failure and/or pulmonary oedema<br>• Known active bleeding or bleeding diatheses<br>• Known active infection requiring antibiotic treatment<br>• Severe hematologic abnormalities (haematocrit <30% and platelet cell count of <100 × 10^3/µL and white blood cell count <4 × 10^3/µL)<br>• Known malignancies requiring active treatment or follow up<br>• Known heart failure with LV EF< 35%<br>• Hypotension (Systolic BP <100mm Hg, DBP<50mmHg)<br>• Uncontrolled hypertension (>160/100mmHg)<br>• History of recurrent syncope suggestive of arrhythmia syncope<br>• Known hepatic failure or abnormal LFTs at baseline (ALT > 2 x ULN, TBL > 1.5 x ULN and ALP > 1.5 x ULN)<br>• Acute kidney injury or chronic kidney disease at Stage > 3B (eGFR < 45 ml/min/1.73m^2)<br>• Acute respiratory failure<br>• Known hyper- or hypothyroidism<br>• History of drug induced Stevens Johnson syndrome, Drug reaction with eosinophilia and systemic symptoms (DRESS syndrome) or toxic epidermal necrolysis<br>• History of recurrent epileptic seizures in the previous 4 years<br>• Average corrected QT interval > 450 msecs<br>• Known chronic active hepatitis (B or C) or HIV infection<br>• Known autoimmune disease requiring active immunosuppressive therapy<br>• History of organ transplantation<br>• Any oral or intravenous immunosuppressive treatment<br>• Known pregnancy at screening or on-going lactation<br>• Current participation in other interventional clinical trials<br>• Contra indication or hypersensitivity to IL-2 treatment

For peer review only

See TIFF Figure 1

Figure 1. Trial design per patient. Each patient will make a total of eight study visits.

See TIFF Figure 2

Figure 2. Trial design for each group in Part A. There are a total of 5 dose levels in Part A.

For peer review only - http://bmjopen.bmj.com/site/about/guidelines.xhtml

See TIFF Figure 3

Figure 3. Trial design for each group in Part B. There are a total of 4 dose levels in Part B

Table 2. **Objective stopping criteria triggering a DMC safety review of dose escalation**

QTcB > 500 msecs (or > 530 msecs if baseline QTcB = 450-480 msecs) OR QTcB change from baseline > 60 msecs (based on an average of triplicate ECGs)
Acute Pulmonary oedema or congestive heart failure
Symptomatic systolic BP < 90 mmHg and/or diastolic BP < 60 mmHg OR persistent symptomatic systolic BP 80-90 mmHg for > 15 mins OR severe hypertension (as defined by BP > 180/120 mmHg)
STEMI occurrence
Atrial fibrillation with rapid ventricular response > 150/min, supraventricular tachycardia or bradycardia that requires treatment or is recurrent or persistent
Sustained ventricular tachycardia or ventricular fibrillation
Any patient who develops doubling of creatinine
Systemic hypersensitivity reaction which cannot be attributed to an identifiable cause
If a life-threatening infection is confirmed clinically with a positive microbiological test
Signs suggestive of hepatic failure including encephalopathy, increasing ascites, signs of coagulopathy, liver pain and/or tenderness on palpation, hypoglycaemia presumed to be secondary to liver failure, active GI bleeding. Withdrawal also if ALT >3 ULN
Seizure activity, coma, severe lethargy or somnolence
Risk of respiratory insufficiency requiring intubation

For peer review only

For peer review only - http://bmjopen.bmj.com/site/about/guidelines.xhtml

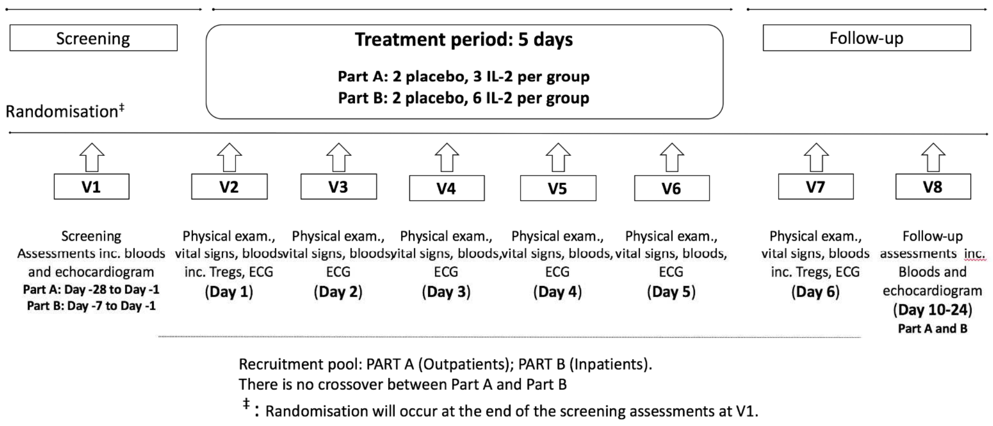

Figure 1. Trial design per patient. Each patient will make a total of eight study visits.

438x188mm (300 x 300 DPI)

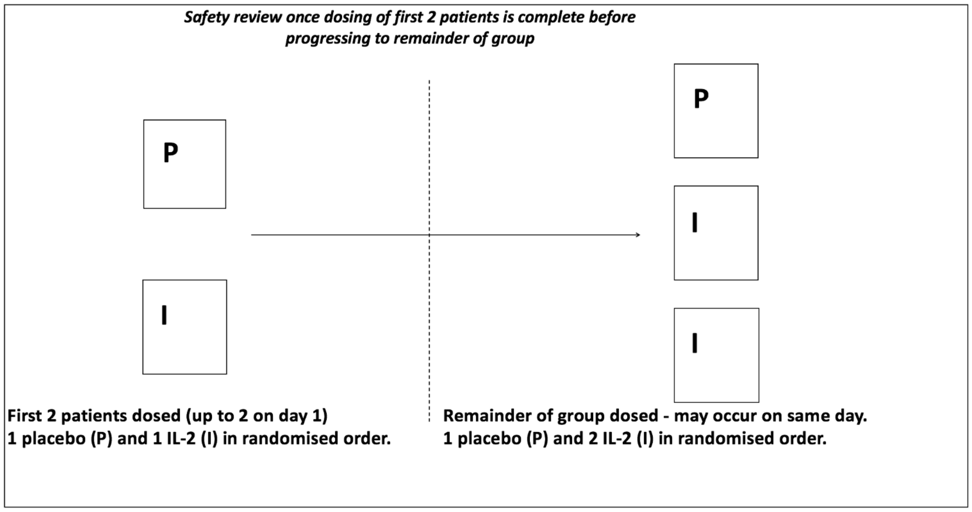

Figure 2. Trial design for each group in Part A. There are a total of 5 dose levels in Part A.

400x214mm (300 x 300 DPI)

For peer review only - http://bmjopen.bmj.com/site/about/guidelines.xhtml

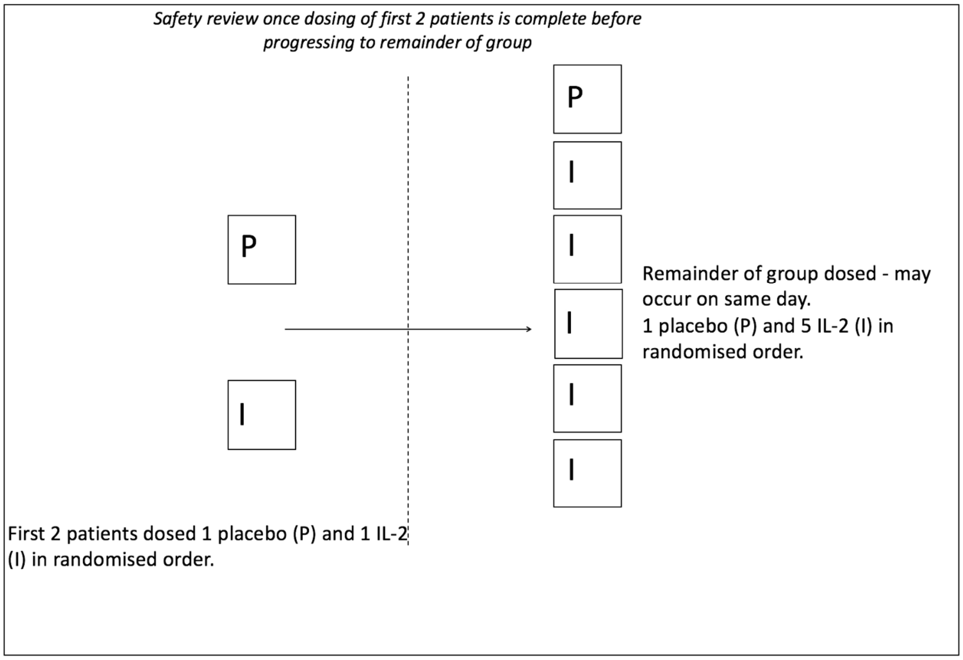

Figure 3. Trial design for each group in Part B. There are a total of 4 dose levels in Part B

329x219mm (300 x 300 DPI)

BMJ Open

Low dose interleukin-2 in patients with stable ischaemic heart disease and acute coronary syndromes (LILACS): protocol and study rationale for a randomised, double-blind, placebo controlled, phase I/II clinical trial

Journal:	*BMJ Open*
Manuscript ID	bmjopen-2018-022452.R1
Article Type:	Protocol
Date Submitted by the Author:	06-Jun-2018

Complete List of Authors:	Zhao, Tian; University of Cambridge Medicine, Division of Cardiovascular Medicine<br>Kostapanos, Michalis; University of Cambridge Medicine, Experimental Medicine and Immunotherapeutics (EMIT) Division<br>Griffiths, Charmaine; Addenbrooke's Hospital, Clinical Trials Unit<br>Arbon, Emma; Addenbrooke's Hospital, Clinical Trials Unit<br>Hubsch, Annette; University of Cambridge Medicine, Experimental Medicine and Immunotherapeutics (EMIT) Division<br>Kaloyirou, Fotini; University of Cambridge Medicine, Experimental Medicine and Immunotherapeutics (EMIT) Division<br>Helmy, Joanna; University of Cambridge Medicine, Experimental Medicine and Immunotherapeutics (EMIT) Division<br>Hoole, Stephen; Papworth Hospital NHS Foundation Trust, Cardiology<br>Rudd, James; University of Cambridge Medicine, Division of Cardiovascular Medicine<br>Wood, Graham; Addenbrooke's Hospital<br>Burling, Keith; Cambridge University Hospitals NHS Foundation Trust, Clinical Biochemistry<br>Bond, Simon; Cambridge University Hospitals NHS Foundation Trust, Cambridge clinical trials unit<br>Cheriyan, Joseph; University of Cambridge Medicine, Division of Experimental Medicine and Immunotherapeutics<br>Mallat, Ziad; University of Cambridge Medicine, Division of Cardiovascular Medicine

**Primary Subject Heading**:	Cardiovascular medicine
Secondary Subject Heading:	Immunology (including allergy), Pharmacology and therapeutics
Keywords:	Ischaemic heart disease < CARDIOLOGY, acute coronary syndrome, interleukin 2, regulatory T cells, Aldesleukin

SCHOLARONE™ Manuscripts

For peer review only

Low dose interleukin-2 in patients with stable ischaemic heart disease and acute coronary syndromes (LILACS): protocol and study rationale for a randomised, double-blind, placebo controlled, phase I/II clinical trial

Tian X. Zhao[1,2], Michalis Kostapanos[2], Charmaine Griffiths[3], Emma L. Arbon[3], Annette Hubsch[2], Fotini Kaloyirou[2], Joanna Helmy[2], Stephen P. Hoole[4], James H.F. Rudd[1], Graham Wood[5], Keith Burling[6], Simon Bond[3], Joseph Cheriyan[2,3*], Ziad Mallat[1*]

[1] Department of Medicine, Division of Cardiovascular Medicine, University of Cambridge, Cambridge, UK
[2] Division of Experimental Medicine and Immunotherapeutics (EMIT), Department of Medicine, University of Cambridge, Cambridge, UK
[3] Cambridge Clinical Trials Unit, Cambridge University Hospitals, Cambridge, UK
[4] Department of Interventional Cardiology, Royal Papworth Hospital NHS Trust, Cambridge, UK
[5] Department of Immunology, Cambridge University Hospitals, Cambridge, UK
[6] Clinical Biochemistry, Cambridge University Hospitals, Cambridge, UK

*Joint senior authors

Correspondence to: Tian Zhao (txz20@cam.ac.uk)

Word count: 5660

For peer review only

Abstract

Introduction:

Inflammation and dysregulated immune responses play a crucial role in atherosclerosis, underlying ischaemic heart disease (IHD) and acute coronary syndromes (ACS). Immune responses are also major determinants of the post-ischaemic injury in myocardial infarction. Regulatory T cells ($CD4^+CD25^+FOXP3^+$; Treg) induce immune tolerance and preserve immune homeostasis. Recent *in vivo* studies suggested that low-dose interleukin-2 (IL-2) can increase Treg cell numbers. Aldesleukin is a human recombinant form of IL-2 which has been used therapeutically in several autoimmune diseases. However, its safety and efficacy is unknown in the setting of coronary artery disease.

Method and analysis:

Low-dose interleukin-2 in patients with stable ischaemic heart disease and acute coronary syndromes (LILACS) is a single centre, first-in-class, dose escalation, two-part clinical trial. Patients with stable IHD (Part A) and ACS (Part B) will be randomised to receive either IL-2 (Aldesleukin; dose range 0.3 – 3 $x10^6$ IU) or placebo once daily, given subcutaneously, for 5 consecutive days. Part A will have 5 dose levels with 5 patients in each group. Group 1 will receive a dose of 0.3 $x10^6$ IU whilst the dose for the remaining 4 groups will be determined upon completion of the preceding group. Part B will have 4 dose levels with 8 patients in each group. The dose of the first group will be based on Part A. Doses for each of the subsequent 3 groups will similarly be determined after completion of the previous group. The primary endpoint is safety and tolerability of aldesleukin, and to determine the dose which increases mean circulating Treg levels by at least 75%.

Ethics and dissemination:

The study recieved a favourable opinion by the Greater Manchester Central Research Ethics Committee, UK (17/NW/0012). The results of this study will be reported, through peer-reviewed journals, conference presentations and an internal organisational report.

Trial registration number: Clinicaltrials.gov (NCT03113773)

Article summary

Strengths and limitations of this study

- The double-blind, placebo-controlled design will allow assessment of the safety and efficacy of low-dose IL-2 in patients with stable ischaemic heart disease and in acute coronary syndrome patients, both conditions where it is currently contraindicated
- The adaptive dose design of this study will allow assessment of the potential for low-dose IL-2 to increase mean circulating Treg levels by at least 75%
- Due to its early phase design, this study is not powered to assess any clinical outcome data for patients

Keywords: ischaemic heart disease, acute coronary syndrome, interleukin-2, regulatory T cells, Aldesleukin

For peer review only

Background

Despite major advances in the treatment, ischaemic heart disease (IHD) remains a significant cause of mortality and morbidity. It is now firmly established that inflammation and the immune response are crucial to the pathophysiology of IHD. This is true both in atherosclerosis which underlies stable angina and in progression to plaque instability and disruption in acute coronary syndrome (ACS) [1].

Although the innate immune system has been better studied in atherosclerosis, the role of the adaptive immune responses is now being increasingly understood. Several studies reported a perturbation of the T cell repertoire in ACS patients [2] with expansion of an effector and activated T cell subset [3], which is, at least in part, directed to antigens contained in the disrupted plaque [4]. Initial pre-clinical findings have shown that regulatory T (Treg) cell mediated immunity reduces experimental atherosclerosis and plaque inflammation [5].

Even though Treg cells in humans are less distinct and more heterogeneous then in mice [6], there is evidence to demonstrate their role in IHD. In ACS patients, there is an imbalance between T effector and Treg cells. Despite the effector T cell compartment activation, the percentage and function of circulating Tregs appear to be significantly decreased in in the setting of ACS [7-10]. This imbalance is thought to play a key role in coronary plaque progression and destabilisation. In this context, low levels of circulating baseline $CD4^+Foxp3^+$ Treg cells were associated with an increased risk for acute coronary events in the Malmö Diet and Cancer Study [11].

Following myocardial infarction, the ischaemic and necrotic myocardial tissue may present self-antigens to the immune system, leading to antigen-specific, autoimmune adaptive responses [12,13]. Recent studies indicate that $CD4^+$ T cells, and particularly Treg cells, are important for the control of post-ischaemic immune responses and the promotion of myocardial healing [13-16]. In another study, inhibition of Treg recruitment to the site of myocardial injury resulted in excessive post-ischaemic inflammation, matrix degradation and adverse remodelling [14]. In contrast, *in vivo* expansion of Treg cells or their therapeutic activation by superagonistic anti-CD28 antibodies attenuated left ventricular remodelling and improved cardiac function [15,16].

Interleukin-2 (IL-2) plays a key role in Treg cell development, expansion, survival and suppressive function [17,18]. Deficiency of either IL-2 or IL-2 receptor in mice greatly compromises Treg development and promotes autoimmune responses [19]. Supplementation of IL-2 substantially increases Treg cell levels and significantly limits plaque development and inflammation in mice prone to atherosclerosis [20,21]. Treg cells show a much lower threshold response to IL-2 receptor signalling compared to effector T cells. This led to the hypothesis that, in contrast to high dose IL-2 designed to activate T effector cells in cancer, supplementation with low doses of IL-2 in the setting of T cell-mediated immune diseases could selectively promote the expansion of Treg cells at the expense of T effector cells, thereby limiting harmful immune responses. This hypothesis was initially confirmed in two pilot human clinical studies in two different disease settings, graft-versus-host disease [22,23] and in hepatitis C virus-induced vasculitis [24]. In both studies, administration of low

For peer review only - http://bmjopen.bmj.com/site/about/guidelines.xhtml

doses of IL-2 in the form of Aldesleukin (daily administration of $0.3x10^6$ to 3.0 $x10^6$ IU IL-2 per square meter of body-surface area for 8 weeks, or repetitive 5-day courses of 1.0 $x10^6$ to 3.0 $x10^6$ IU IL-2) led to a rapid and marked expansion of the circulating pool of Treg cells, which were at least doubled without affecting the pool of conventional $CD4^+$ T (i.e. T effector) cells. The expanded Tregs retained potent suppressive functions and the treatment was associated with a reduction in the inflammatory response and a concomitant clinical improvement in a substantial proportion of patients. Treatment with low dose IL-2 was safe and no adverse effects were reported. This strategy is currently being adapted and tested in various disease settings, where Treg cell promotion is believed to be of potential therapeutic benefit [23-26]. In this trial, we hypothesise that low dose IL-2 (Aldesleukin) can be used in IHD to increase Treg numbers and to rebalance the immune system with the overall goal of decreasing recurrent myocardial infarction and cardiovascular death.

Aldesleukin (Proleukin®, Novartis) is a commercially available IL-2 licensed for the treatment of metastatic renal cell carcinoma in the UK. It is produced by recombinant DNA technology using an Escherichia coli strain, which contains a genetically engineered modification of the human IL-2 gene, and is administered either intravenously (IV) or subcutaneously (SC). Following short IV infusion, its pharmacokinetic profile is typified by high plasma concentrations, rapid distribution into the extravascular space and a rapid renal clearance. The recommended doses for continuous infusion and subcutaneous injection (as detailed in the Summary of Product Characteristics) are repeated cycles of 18 x 10^6 IU per m^2 per 24-hours for 5 days, and repeated doses of 18 x 10^6 IU respectively. Peak plasma levels are reached in 2-6 hours after subcutaneous administration, with bioavailability of Aldesleukin ranging between 31–47%. The process of absorption and elimination of subcutaneous Aldesleukin is described by a one-compartment model, with a 45-minute absorption half-life and a 3-5 hour elimination half-life [27].

Use of IL-2 in clinical trials to date

The first report of effective IL-2 therapy in human cancer trials was published in 1985 [28]. The trial patients in that study were placed on dose-escalated IL-2 regimens, of up to approximately 120 Million IU (MIU). Associated with these high IL-2 doses were side effects such as capillary leak syndrome (which is characterized by a loss of vascular tone and extravasation of plasma proteins and fluid into the extravascular space, ultimately resulting in hypotension, tachycardia, dyspnoea and pulmonary oedema), and kidney and liver damage (both characterised by increased serum creatinine and bilirubin levels respectively) [29].

The use of low dose IL-2 to expand Treg cell populations in autoimmune and allo-inflammatory conditions has been previously explored and published in human clinical trials. In these studies, patients received at least 1 dose of IL-2 ranging from $0.3x10^6$ IU – $3.0x10^6$ IU. In two studies of 12 and 21 healthy volunteers respectively, there were minimal adverse events (AEs), consisting mainly of grade 1 injection site reactions. No cardiovascular AEs were noted [30,31]. In one phase 1/2a study, 24 patients with diabetes mellitus were recruited and given a maximum dose of $3.0x10^6$ IU daily for 5 days. The authors found that IL-2 was well tolerated at all doses, with no serious adverse events (SAEs). However, there was a dose-response relationship for non-serious AEs. The most common AEs in the

treatment phase were injection-site reactions and an influenza-like syndrome [32]. In a later trial of 40 type 1 diabetics, the authors found that doses of Aldesleukin were well tolerated at all doses, with no serious adverse events (SAEs) reported. The majority of participants had an expected AE at the injection site consisting a non-itchy, local (1–5 cm), non-painful erythematous rash which resolved on average by day 10 [33]. No cardiovascular AEs were reported in either study. Low dose IL-2 has also been used in 38 SLE patients[26], who are considered to have a higher risk of coronary artery disease and therefore cardiac events [34]. However, no SAEs were observed whilst injection site reactions and flu like symptoms were observed in 13.2% and 5.3% of patient respectively [26].

Nevertheless, IL-2 is contraindicated in patients with a significant history, or current evidence of, severe cardiac disease. Therefore, we sought to determine the safety and efficacy of low dose Aldesleukin in patients with pre-existing cardiac conditions. A detailed and conservative risk mitigation strategy was adopted to ensure patient safety was maintained throughout the conduct of the trial (see Supplementary File 1). We hypothesise that low dose IL-2, unlike higher doses, can be safely administered and is effective in expanding the Treg population in patients with stable and acute coronary artery disease. In this trial, low dose IL-2 will initially be administered in stable IHD patients at escalating doses and, following safety reviews, will be given to ACS patients. The Treg response data from the ACS population will help select the most appropriate dose to assess efficacy in future clinical trials in ACS.

Method

This is a an academically driven, prospective single centre, randomised, double-blind, placebo controlled, phase 1/2 clinical trial. It will be performed at the National Institute for Health Research/Wellcome Trust Cambridge Clinical Research Centre, Cambridge University Hospitals, Cambridge, UK with overall study co-ordination provided by the Cardiovascular Trials Office of the of the Cambridge Clinical Trials Unit, Cambridge University Hospitals NHS Foundation Trust. The study is sponsored by Cambridge University Hospitals NHS Foundation Trust.

Study populations

The trial will be performed in two parts. Part A will include patients with stable IHD aged 18-75 years with a clinical diagnosis of IHD for more than 6 months (ascertained by either having had a previous diagnosis of MI or having symptoms of angina and a coronary angiogram showing obstructive (stenosis >50%) coronary disease). The inclusion and exclusion criteria are detailed in Table 1, but in summary, patients with a myocardial infarction within the last 6 months, cardiogenic shock, hypo/hypertension, heart failure (EF<45%), pro-arrhythmogenic conditions, renal, hepatic, thyroid or haematological dysfunction, active infection, poorly controlled diabetes, active autoimmune disease, current malignancy, history of seizures or immunosuppression will be excluded from this part of the study.

For peer review only

Part B will be performed after Part A and will include patients aged of 18-85 years admitted with a diagnosis of non-ST elevation myocardial infarction. The inclusion and exclusion criteria are detailed in Table 1. In brief, patients with cardiogenic shock, hyper/hypotension, heart failure (ejection fraction<35%), long QT or arrhythmias, renal, hepatic, thyroid or haematological dysfunction, active infection, active autoimmune disease, current malignancy, history of seizures or immunosuppression will be excluded from this part of the study.

Study protocol

Part A

Patients will be recruited from advertisements, outpatient clinics or research databases. Participants will have at least 24 hours to review the Patient Information Sheet prior to informed consent. Study procedures will only be conducted following formal written consent at the screening visit 1 (V1). Baseline blood tests will consist of electrolytes, renal, liver and thyroid function, full blood count, clotting profile, Hepatitis B/C and HIV screening, HbA1c, and pregnancy screen (where applicable). Baseline vitals, electrocardiogram (ECG), echocardiogram, clinical history and physical examination will also be performed. Randomisation will be carried out via a paper based concealment list generated by a statistician. To maintain the overall quality and legitimacy of the clinical trial, unblinding will only occur in exceptional circumstances when knowledge of the actual treatment is essential for further clinical management of the patient.

The trial design is described in detail in Figure 1. In brief, following randomisation at V1, patients will attend 5 consecutive daily outpatient visits (V2-6) during which blinded subcutaneous injections of Aldesleukin or placebo will be administered. At each visit, prior to the study drug administration, the medical history will be obtained/reviewed, along with a physical examination, baseline vitals, safety bloods and a 12 lead-ECG. Patients will have continuous cardiac monitoring during each visit for at least 30minutes pre- and 1.5 hours post-dose. After dosing, a series of ECGs will be performed at 15 minutes, 30 minutes and 60 minutes whilst vitals will be assessed at 30 minutes and 60 minutes. For each dosing visit, serum IL-2 levels will be taken at baseline and at 90 minutes post-dose.

There are 2 follow up visits (V7 and V8). Assessments during both visits will include a medical review, physical examination, vitals, ECGs and safety bloods tests. Additionally, at V8, a follow up thyroid function blood test and echocardiogram will be performed.

In addition, during visits V2, V7 (Figure 1), cardiac biomarkers (high sensitivity C-reactive protein (hs-CRP), IL-6, brain-type natriuretic peptide (BNP) and troponin) and lymphocyte subsets (including Treg level, see below) analysis will be performed. During visit V8, cardiac biomarkers will be re-assessed.

A total of 25 patients, 5 in each of the 5 dosing groups, will be included in Part A (drug:placebo ratio of 3:2). In line with current Phase 1 trial designs, a sentinel dosing approach will be employed whereby the first 2 patients of each group will be allocated to either Aldesleukin or placebo in a random order. After a blinded safety review of these first 2 patients, the remaining patients will then be dosed (see Figure 2).

For peer review only - http://bmjopen.bmj.com/site/about/guidelines.xhtml

Part B

Patients admitted with a primary diagnosis of non-ST elevation myocardial infarction will be recruited from the medical and cardiology wards at Cambridge University Hospitals NHS Foundation Trust. Patients may continue to receive trial treatments if they are transferred to the local interventional centre at Royal Papworth Hospital. Participants will be given at least 24 hours to review the Patient Information Sheet prior to formal consent. Dosing should commence within 8 days of screening. All visits and blinding procedures will be the same as Part A. However, in part B, a total of 32 patients will be recruited, 8 patients in each of the 4 dosing groups (drug:placebo ratio of 6:2). A sentinel approach to dosing will also be employed in each group. After a blinded safety review of the first 2 patients, the remaining cohort will be randomly allocated to study treatments as shown in Figure 3. The visit schedule for each patient is the same as Part A (Figure 1).

Dose escalation strategy

The first group of patients in Part A will receive $0.3x10^6$ IU of Aldesleukin daily. Thereafter, a blinded review of patient data by the blinded Trial Management Group (TMG) including review of adverse events, blood results, ECGs, clinical records and where possible, drug pharmacodynamics and pharmacokinetics. The TMG will comprise of an experienced, accredited early phase lead physician (LILACS Chief Investigator), a research physician/scientist, a research nurse, trial co-ordinator and an unblinded study statistician (for the purposes of data analysis). All data presented by the unblinded statistician to the TMG will be in an aggregated format, to preserve the blind for the other TMG members. This is consistent with the commercial standard for such early phase trials in industry. . The dose in the second group will be determined after this review and the same process will be followed in each of the following groups. The maximum dose increments allowed by the protocol between groups will be double the previous dose, and capped to a maximum 3.0 $x10^6$ IU.

A robust set of specific and general withdrawal criteria, as well as objective stopping criteria have been put into place to maintain the safety:risk benefit, in particular due to the risk of capillary leak syndrome .

Following completion of Part A, an unblinded independent Data Monitoring Committee (DMC) will review all available safety data, together with any other analyses that the committee may request. The DMC will be comprised of an independent group of clinical researchers with suitable experience in experimental medicine and early phase clinical trials. These researchers are independent from the trial team and have not been involved in the setup or running of this clinical trial. They will determine whether it is safe to progress to Part B, based on available safety and pharmacodynamic data provided by the unblinded statistician. After this analysis, the dose in each group will be determined based on the review of ongoing patient data by the Trial Management Group, as in Part A previously. The protocol mandates that the maximum dose used in Part B will not exceed that of Part A.

For peer review only - http://bmjopen.bmj.com/site/about/guidelines.xhtml

DMC will be governed by a charter set up *a priori*, and signed up by all members prior to the commencement of the trial (see Supplemental File 2).

Outcome measure

Part A

The primary outcome will be the safety of IL-2 in patients. This will be assessed through:

- A review of AEs and SAEs, and concomitant medications
- Changes in safety bloods (electrolytes (sodium, potassium, urea), bone profile (calcium, phosphate), serum creatinine, liver function tests (alanine transaminase, aspartate transaminase, alkaline phosphatase, bilirubin, gamma GT), thyroid function tests (thyroid stimulating hormone), blood glucose, full blood count and differential, clotting (prothrombin time, activated partial thromboplastin time)
- 12-Lead ECG and cardiac monitoring changes (arrhythmias, ischaemic changes, QTcB)
- Vital observations (blood pressure, heart rate, respiratory rate, peripheral oxygen saturation, temperature)
- Echocardiogram changes at baseline and follow up

Exploratory endpoints will include:

- Change in the mean circulating Treg level measured by fluorescence activated cell sorting (FACS) analysis following treatment with IL-2, over the 5 days of the treatment period.
- Change in cardiac biomarker measurements including hs-CRP, troponin I, IL-6 and b-type natriuretic peptide) from analysed blood samples.
- Change in lymphocyte subsets measured by FACS analysis
- Pharmacokinetic analysis of IL-2 levels

Part B

As with Part A, the primary endpoint will be safety and tolerability of IL-2. A further primary endpoint will be the change in mean circulating Treg levels and whether IL-2 increases mean circulating Treg levels by at least 75% over the 5 days of the treatment period. Exploratory endpoints are the same as for Part A.

Adverse event reporting

Adverse events (AEs), adverse reactions (ARs), severe AE/ARs (SAEs/ SARs) and suspected unexpected SARs (SUSARs) will be defined as per the International Conference on Harmonisation definitions. A suitably qualified medical doctor will determine the relationship and causality of each AE to the study drug as either 'related' (defined as having a plausible temporal relation and not judged attributable to other causes) or 'not related'. They will also make an assessment on severity and seriousness. Abnormal or significant changes in laboratory results are only AEs if they are deemed to be of clinical significance or if a medical intervention is required. Whether a patient needs to be withdrawn due to the severity of their AE is left up to the discretion of principal investigator.

For peer review only - http://bmjopen.bmj.com/site/about/guidelines.xhtml

Lymphocyte analysis

Lymphocyte subset analysis will be performed at the Department of Clinical Immunology, Cambridge University Hospitals, Cambridge, UK, within 4 hours of sample collection in EDTA. Laboratory technicians will be blinded to treatment allocation. The antibodies that will be used are anti-CD3 (clone SK7, phycoerythrin [PE]-Cy7-labelled; BD Biosciences), anti-CD4 (clone RPA-T4, FITC-labelled; BD Biosciences), anti-CD127 (clone HIL-7R-M21, PE-labelled; BD Biosciences), anti-CD25 (clone M-A251 and 2A3, allophycocyanin [APC]-labelled; BD Biosciences), anti-CD45RA (clone HI100, APC-Cy7-labelled; BioLegend), and anti-CD62L (clone DREG-56, PerCP/Cy5.5-labelled; BioLegend). Whole blood will be assessed by performing clinical FACS to measure the absolute lymphocyte count, lymphocytes subsets, and CD25 expression on Treg cells. Fixed concentrations of fluorescently labelled beads will be added to whole blood to count the absolute number of lymphocytes, $CD3^+$, $CD4^+$ and $CD8^+$ T cells, $CD19^+$ B cells and $CD19^-$ $CD16^+$ $CD56^+$ NK. Simultaneous whole blood FACS assay will be performed where a lymphocyte gate is drawn to include all events whilst the $CD3^+$, $CD4^+$ T-cell gate excludes $CD8^+$ T cells and B cells. Six standardised beads labelled with different quantities of fluorescent allophycocyanin are measured by FACS to accurately measure CD25-APC on the surface of Tregs compared with a standardised curve. To minimise interassay variation, mean fluorescence intensity can be read from this curve. Tregs will be defined by $CD3^+CD4^+CD25^{high}CD127^{low}$ makers and will be separated from non-Tregs and used to calculate the absolute Treg count out of $CD3^+CD4^+$ T cells. Among the non-Treg T effector $CD43^+CD4^+$ population, we will define effector memory cells by $CD45RA^-CD62L^-$ markers, effector memory CD45 RA^+(TEMRA) cells by $CD45RA^+CD62L^-$ markers, naïve effectors cells by $CD45RA^+CD62L^+$ markers and central memory cells by $CD45RA^-CD62L^+$ markers . Total memory effectors are the sum of central memory and effector memory cells.

Cardiac biomarkers

Blood will be taken in gel serum tubes and the serum will be banked and analysed at the Core Biochemical Assay Laboratory, Cambridge. Hs-CRP and NT-proBNP will be measured by immunoassays on the Siemens Dimension EXL autoanalyser. All reagents and calibrators are supplied by Siemens and assays will be performed according to the manufacturer's instructions.

IL-6 will be measured in duplicate using ultra-sensitive electrochemical luminescence immunoassay on the Mesoscale Discovery assay platform and read on the MesoScale Diagnostics Sector Imager 6000. All reagents and calibrators will be supplied by MesoScale Discovery.

Stopping criteria

Dose escalation stopping criteria will be met if 2 patients within a trial group experience any combination of: a SAE defined as possibly, probably or definitely related to the trial drug

For peer review only - http://bmjopen.bmj.com/site/about/guidelines.xhtml

(i.e. it is a SAR), an adverse event that is severe and at least possibly related to the trial drug, or any of the objective stopping criteria detailed Table 2. The following will then occur:

- Dosing will be immediately discontinued for the patients experiencing the event
- Dosing will be halted for all other patients currently in the treatment period of the trial (i.e. patients receiving treatment in the same group)
- A safety review by the independent Data Monitoring Committee (DMC) will be conducted to determine how to proceed with the trial
- Any further single instances of the events outlined above for the same group will trigger a further DMC safety review
- Any patients who have their dosing discontinued will be withdrawn from the trial

Additionally, specific objective stopping criteria are set out in Table 2 which may trigger an unscheduled DMC review prior to any further dose escalations. Specific and general withdrawal criteria are also listed in Supplementary File 1.

Safety monitoring committees

The TMG and the DMC are independent of each other. The function of the DMC is delineated in the DMC charter (Supplemental File 2). The Chief Investigator of the trial will report to the DMC on the course of the trial during open sessions of the DMC. All DMC meetings will be held in private without any involvement of the trial team. The unblinded statistician is the only person who reports to the DMC and is part of the TMG. However, any data presented to the TMG is presented in a manner that maintains the blind.

The TMG will assess safety in a blinded manner on an ongoing basis at regular intervals during the course of the trial. Between Part A and Part B, the DMC will be formally convened to review the unblinded data with the unblinded statistician to determine whether it is reasonable for the trial to progress to the next stage.

Statistical methods and data handling

This is an exploratory study that is not designed to formally test a hypothesis in a confirmatory fashion. Given that both parts of the trial have clinical safety as primary endpoints, a formal power calculation is not relevant. A sample size of 57 patients is achievable within the proposed time scale, given the size of the targeted patient population at our study site. The frequency of adverse events per patient will be summarised for each event based on dose level. Summary statistics of laboratory values by dose and visit will be produced where required. The statistician will use the data from each group to perform a modelling analysis of the effect of Aldesleukin based on dose and effect size using a smoothed line plot of the mean and 95% confidence internals. Generally summary statistics of continuous variables will report mean, median, SD, min and max, although a log-transformed scale may be used where the data are skewed. Binary or categorical variables will be summarised using the p% (x/n) format. The Treg data and other secondary biomarker endpoints will be summarised with individual patient profiles over time, and summary statistics broken down by dose and visit. Formal estimates of the differences

For peer review only - http://bmjopen.bmj.com/site/about/guidelines.xhtml

between doses will be made at each time point with accompanying 95% confidence intervals and p-values.

Subjects will be coded by a numeric code to create an anonymous dataset. All data will be transferred into a Case Report Form, which will be coded onto a MACRO database. All data will be anonymised and stored encrypted on a secured computer to ensure patient confidentiality.

Patient and public involvement

Heart attacks are distressing and impacts patients' lives dramatically [35]. The aim of this research is to help ameliorate this issue and potentially reduce reoccurrence. Lay members of the ethics committee reviewed this study and made constructive comments which have been addressed. Patients were not involved in the recruitment to or conduct of the study. All patients provided full informed consent, with at least 24 hours to consider the information and at least two opportunities to discuss the trial in detail with the investigators. The results of the study will be disseminated to all study patients at the end of the trial.

Study timeline

The trial began on the 15th of May 2017. The anticipated final follow-up visit(s) will be in January 2019. Primary analyses are projected to be completed by February 2019.

Ethics and dissemination

The study was given a favourable opinion by the Greater Manchester Central Research Ethics Committee, UK (17/NW/0012), and approved by the UK’s Health Research Authority. The MHRA formally granted regulatory acceptance on 28th April 2017. All study procedures will be conducted after formal written consent, in accordance with the Declaration of Helsinki. The trial was registered on clinicaltrials.gov (NCT03113773) prior to trial commencement, and the results of this study will be published in a peer reviewed journal after completion.

Funding statement and acknowledgements

This work was funded by the Medical Research Council, grant number MR/N028015/1 and the British Heart Foundation Cambridge Centre of Excellence (RCAG/521). Setup and running of the study and decision to publish results is independent of the funder. JC acknowledges funding support from the National Institute for Health Research Cambridge Comprehensive Biomedical Research Centre. ZM acknowledges funding support from the British Heart Foundation for his BHF Chair. JHFR acknowledges funding support from the HEFCE, the NIHR Cambridge Biomedical Research Centre, the British Heart Foundation, the EPSRC and the Wellcome Trust. We acknowledge support from the Cambridge Clinical Research Centre and the Core Biochemistry Assay Laboratory in the conduct of this trial and its endpoints.

For peer review only - http://bmjopen.bmj.com/site/about/guidelines.xhtml

Authors' contributions and competing interests

TZ, MK, SPH, JC and ZM contributed to the writing of this manuscript. TZ, MK, CG, EA, AH, FK, JH, JHFR, GW, KB, SB, JC and ZM contributed to the writing of the protocol. The authors do not report any competing interests.

References

1 Libby P, Ridker PM, Hansson GK, *et al.* Inflammation in atherosclerosis: from pathophysiology to practice. *Journal of the American College of Cardiology* 2009;**54**:2129–38. doi:10.1016/j.jacc.2009.09.009

2 Liuzzo G, Biasucci LM, Trotta G, *et al.* Unusual CD4+CD28nullT Lymphocytes and Recurrence of Acute Coronary Events. *Journal of the American College of Cardiology* 2007;**50**:1450–8. doi:10.1016/j.jacc.2007.06.040

3 Ammirati E, Vermi AC, Cianflone D, *et al.* Expansion of T-Cell Receptor dim Effector T Cells in Acute Coronary Syndromes. *Arteriosclerosis, Thrombosis, and Vascular Biology* 2008;**28**:2305–11. doi:10.1161/ATVBAHA.108.174144

4 Caligiuri G, Paulsson G, Nicoletti A, *et al.* Evidence for antigen-driven T-cell response in unstable angina. *Circulation* 2000;**102**:1114–9.

5 Ait-Oufella H, Salomon BL, Potteaux S, *et al.* Natural regulatory T cells control the development of atherosclerosis in mice. *Nature Medicine* 2006;**12**:178–80. doi:10.1038/nm1343

6 Shevach EM. Biological functions of regulatory T cells. *Adv Immunol* 2011;**112**:137–76. doi:10.1016/B978-0-12-387827-4.00004-8

7 Mor A, Luboshits G, Planer D, *et al.* Altered status of CD4+CD25+ regulatory T cells in patients with acute coronary syndromes. *European Heart Journal* 2006;**27**:2530–7. doi:10.1093/eurheartj/ehl222

8 Han S-F, Liu P, Zhang W, *et al.* The opposite-direction modulation of CD4+CD25+ Tregs and T helper 1 cells in acute coronary syndromes. *Clinical Immunology* 2007;**124**:90–7. doi:10.1016/j.clim.2007.03.546

9 Cheng X, Yu X, Ding Y-J, *et al.* The Th17/Treg imbalance in patients with acute coronary syndrome. *Clinical Immunology* 2008;**127**:89–97. doi:10.1016/j.clim.2008.01.009

10 Ammirati E, Cianflone D, Banfi M, *et al.* Circulating CD4+CD25hiCD127lo Regulatory T-Cell Levels Do Not Reflect the Extent or Severity of Carotid and Coronary Atherosclerosis. *Arteriosclerosis, Thrombosis, and Vascular Biology* 2010;**30**:1832–41. doi:10.1161/ATVBAHA.110.206813

11 Wigren M, Björkbacka H, Andersson L, *et al.* Low levels of circulating CD4+FoxP3+ T cells are associated with an increased risk for development of myocardial infarction but not for stroke. *Arteriosclerosis, Thrombosis, and Vascular Biology* 2012;**32**:2000–4. doi:10.1161/ATVBAHA.112.251579

12 Zhang M, Alicot EM, Chiu I, *et al.* Identification of the target self-antigens in reperfusion injury. *J Exp Med* 2006;**203**:141–52. doi:10.1084/jem.20050390

13 Hofmann U, Beyersdorf N, Weirather J, *et al.* Activation of CD4+ T lymphocytes improves wound healing and survival after experimental myocardial infarction in mice. *Circulation* 2012;**125**:1652–63. doi:10.1161/CIRCULATIONAHA.111.044164

For peer review only

14 Dobaczewski M, Xia Y, Bujak M, *et al.* CCR5 Signaling Suppresses Inflammation and Reduces Adverse Remodeling of the Infarcted Heart, Mediating Recruitment of Regulatory T Cells. *The American Journal of Pathology* 2010;**176**:2177–87. doi:10.2353/ajpath.2010.090759

15 Matsumoto K, Ogawa M, Suzuki J-I, *et al.* Regulatory T lymphocytes attenuate myocardial infarction-induced ventricular remodeling in mice. *Int Heart J* 2011;**52**:382–7.

16 Tang T-T, Yuan J, Zhu Z-F, *et al.* Regulatory T cells ameliorate cardiac remodeling after myocardial infarction. *Basic Res Cardiol* 2012;**107**:232. doi:10.1007/s00395-011-0232-6

17 Malek TR, Bayer AL. Tolerance, not immunity, crucially depends on IL-2. *Nat Rev Immunol* 2004;**4**:665–74. doi:10.1038/nri1435

18 Wing K, Sakaguchi S. Regulatory T cells exert checks and balances on self tolerance and autoimmunity. *Nat Immunol* 2010;**11**:7–13. doi:10.1038/ni.1818

19 Malek TR. The biology of interleukin-2. *Annu Rev Immunol* 2008;**26**:453–79. doi:10.1146/annurev.immunol.26.021607.090357

20 Foks AC, Frodermann V, Borg ter M, *et al.* Differential effects of regulatory T cells on the initiation and regression of atherosclerosis. *Atherosclerosis* 2011;**218**:53–60. doi:10.1016/j.atherosclerosis.2011.04.029

21 Dinh TN, Kyaw TS, Kanellakis P, *et al.* Cytokine therapy with interleukin-2/anti-interleukin-2 monoclonal antibody complexes expands CD4+CD25+Foxp3+ regulatory T cells and attenuates development and progression of atherosclerosis. *Circulation* 2012;**126**:1256–66. doi:10.1161/CIRCULATIONAHA.112.099044

22 Koreth J, Matsuoka K-I, Kim HT, *et al.* Interleukin-2 and regulatory T cells in graft-versus-host disease. *N Engl J Med* 2011;**365**:2055–66. doi:10.1056/NEJMoa1108188

23 Matsuoka K-I, Koreth J, Kim HT, *et al.* Low-dose interleukin-2 therapy restores regulatory T cell homeostasis in patients with chronic graft-versus-host disease. *Sci Transl Med* 2013;**5**:179ra43–3. doi:10.1126/scitranslmed.3005265

24 Saadoun D, Rosenzwajg M, Joly F, *et al.* Regulatory T-cell responses to low-dose interleukin-2 in HCV-induced vasculitis. *N Engl J Med* 2011;**365**:2067–77. doi:10.1056/NEJMoa1105143

25 Rosenzwajg M, Churlaud G, Mallone R, *et al.* Low-dose interleukin-2 fosters a dose-dependent regulatory T cell tuned milieu in T1D patients. *J Autoimmun* 2015;**58**:48–58. doi:10.1016/j.jaut.2015.01.001

26 He J, Zhang X, Wei Y, *et al.* Low-dose interleukin-2 treatment selectively modulates CD4+ T cell subsets in patients with systemic lupus erythematosus. *Nature Medicine* 2016;**22**:991–3. doi:10.1038/nm.4148

27 Novartis Pharmaceuticals UK Ltd. Proleukin Summary of Product Characteristics Updated 20-Jan-2015. https://www.medicines.org.uk/emc/print-document?documentId=19322

28 Rosenberg SA, Lotze MT, Muul LM, *et al.* Observations on the systemic administration of autologous lymphokine-activated killer cells and recombinant interleukin-2 to patients with metastatic cancer. *N Engl J Med* 1985;**313**:1485–92. doi:10.1056/NEJM198512053132327

29 Aronson FR, Libby P, Brandon EP, *et al.* IL-2 rapidly induces natural killer cell adhesion to human endothelial cells. A potential mechanism for endothelial injury. *J Immunol* 1988;**141**:158–63.

30 Ito S, Battiwalla M, Melenhorst JJ, *et al.* Ultra-Low Dose IL-2 Safely Expands Regulatory T Cells and CD56 NK Cells in Healthy Volunteers: Towards Safer Stem Cell Donors? *Blood* 2015;**120**:3283.

For peer review only - http://bmjopen.bmj.com/site/about/guidelines.xhtml

31 Ito S, Bollard CM, Carlsten M, *et al.* Ultra-low dose interleukin-2 promotes immune-modulating function of regulatory T cells and natural killer cells in healthy volunteers. *Mol Ther* 2014;**22**:1388–95. doi:10.1038/mt.2014.50

32 Hartemann A, Bensimon G, Payan CA, *et al.* Low-dose interleukin 2 in patients with type 1 diabetes: a phase 1/2 randomised, double-blind, placebo-controlled trial. *Lancet Diabetes Endocrinol* 2013;**1**:295–305. doi:10.1016/S2213-8587(13)70113-X

33 Todd JA, Evangelou M, Cutler AJ, *et al.* Regulatory T Cell Responses in Participants with Type 1 Diabetes after a Single Dose of Interleukin-2: A Non-Randomised, Open Label, Adaptive Dose-Finding Trial. *PLoS Med* 2016;**13**:e1002139. doi:10.1371/journal.pmed.1002139

34 Bartels CM, Buhr KA, Goldberg JW, *et al.* Mortality and cardiovascular burden of systemic lupus erythematosus in a US population-based cohort. *J Rheumatol* 2014;**41**:680–7. doi:10.3899/jrheum.130874

35 Grace SL, Abbey SE, Irvine J, *et al.* Prospective examination of anxiety persistence and its relationship to cardiac symptoms and recurrent cardiac events. *Psychother Psychosom* 2004;**73**:344–52. doi:10.1159/000080387

For peer review only

Tables and figure legends

Table 1. Trial inclusion and exclusion criteria for parts A and B

Part A	Inclusion criteria	• Age 18-75 years old inclusive<br>• Previous history (> 6 months from planned first day of dosing) of coronary artery disease<br>• No history of recent (< 6 months) admissions for an unstable cardiovascular event e.g. MI, unstable angina, ACS<br>• Written informed consent for participation in the trial
	Exclusion criteria	• Current presentation with cardiogenic shock (systolic blood pressure <80 mm Hg, unresponsive to fluids, or necessitating catecholamines), severe congestive heart failure and/or pulmonary oedema<br>• Known active bleeding or bleeding diatheses<br>• Known active infection requiring antibiotic treatment<br>• Severe hematologic abnormalities (haematocrit <30% and platelet cell count of $<100 \times 10^3/\mu L$ and white blood cell count $<4 \times 10^3/\mu L$)<br>• Known malignancies requiring active treatment or follow up (However, patients with current/a history of localised basal or squamous cell skin cancer are not excluded from participation in this trial)<br>• Known heart failure with impaired LV function (LV EF <45%)<br>• Hypotension (Systolic BP<100mm Hg, DBP<50mmHg) at screening<br>• Uncontrolled hypertension (>160/100 mmHg) at screening<br>• History of recurrent syncope (Electrocardiographic history suggestive of arrhythmia syncope (e.g. bifascicular block, sinus bradycardia < 40 beats per minute in absence of sinoatrial block or medications, pre-excited QRS complex, abnormal QT interval, ST segment elevation leads V1 through V3 [Brugada syndrome], negative T wave in right precordial leads and epsilon wave [arrhythmogenic right ventricular dysplasia/cardiomyopathy]))<br>• Known hepatic failure or abnormal LFTs at baseline (ALT > 2 x ULN)<br>• Elevated Total Bilirubin Levels, (TBL > 1.5 x ULN) and Alkaline Phosphatase, ALP (ALP > 1.5 x ULN), at baseline<br>• Acute kidney injury or chronic kidney disease at Stage > 3B (eGFR < 45 ml/min/1.73m^2)<br>• Respiratory failure<br>• Known hyper- or hypothyroidism<br>• History of drug induced Stevens Johnson syndrome, Drug reaction with eosinophilia and systemic symptoms (DRESS syndrome) or toxic epidermal necrolysis<br>• History of recurrent epileptic seizures in the previous 4 years; repetitive or difficult to control seizures, coma or toxic psychosis lasting >48 hours<br>• If known diabetic, uncontrolled diabetes defined as HbA1c > 64 mmol/mol<br>• Average corrected QT interval > 450 msecs using Bazett's formula using

For peer review only

		triplicate ECGs (or > 480 msecs if bundle branch block)<br>• Known chronic active hepatitis (B or C)<br>• Known HIV infection<br>• Current infection possibly related to recent or on-going immunosuppressive treatment<br>• Known autoimmune disease requiring active immunosuppressive therapy<br>• History of organ transplantation<br>• Any oral or intravenous Immunosuppressive treatment including Prednisolone, hydrocortisone or disease modifying drugs such as Azathioprine, interferon-alpha, Cyclophosphamide or Mycophenolate. [Other immunosuppressive therapies should be discussed with PI. Inhaled or topical steroids are permissible.]<br>• Known pregnancy at screening or visit 2 (where applicable)<br>• On-going lactation<br>• Inability to comply with trial procedures<br>• Current participation in other interventional clinical trials<br>• Contra indication to IL-2 treatment or hypersensitivity to IL-2 or to any of its excipients<br>• Unwillingness or inability to provide written informed consent for participation<br>• Any medical history or clinically relevant abnormality that is deemed by the principal investigator/delegate and/or medical monitor to make the patient ineligible for inclusion because of a safety concern
Part B	Inclusion criteria	• Age 18-85 years old inclusive<br>• Current admission (on at least screening visit) with acute coronary syndrome (non-ST elevation myocardial infarction, i.e., NSTEMI, or unstable angina) with symptoms of myocardial ischaemia lasting 10 minutes or more with the patient at rest or with minimal effort plus either elevated levels of TnI on admission or dynamic changes in ECG (new ST-T changes) or T-wave inversion<br>• Willingness to be dosed within 8 days from initial date of current admission for ACS<br>• Written informed consent for participation in the trial
	Exclusion criteria	• ST elevation myocardial infarction (heart attack) on this admission.<br>• Current presentation with cardiogenic shock (systolic blood pressure <80 mm Hg, unresponsive to fluids, or necessitating catecholamines), electrical instability, severe congestive heart failure and/or pulmonary oedema<br>• Known active bleeding or bleeding diatheses<br>• Known active infection requiring antibiotic treatment<br>• Severe hematologic abnormalities (haematocrit <30% and platelet cell count of <100 × 10^3/μL and white blood cell count <4 × 10^3/μL)<br>• Known malignancies requiring active treatment or follow up (However, patients with current/a history of localised basal or squamous cell skin

For peer review only - http://bmjopen.bmj.com/site/about/guidelines.xhtml

		cancer are not excluded from participation in this trial)<br>• Known heart failure with impaired LV function with LV EF< 35%<br>• Hypotension (Systolic BP <100mm Hg, DBP<50mmHg)<br>• Uncontrolled hypertension (>160/100mmHg) at screening<br>• History of recurrent syncope (Electrocardiographic history suggestive of arrhythmia syncope (e.g., bifascicular block, sinus bradycardia < 40 beats per minute in absence of sinoatrial block or medications, pre-excited QRS complex, abnormal QT interval, ST segment elevation leads V1 through V3 [Brugada syndrome], negative T wave in right precordial leads and epsilon wave [arrhythmogenic right ventricular dysplasia/cardiomyopathy]))<br>• Known hepatic failure or abnormal LFTs at baseline (ALT > 2 x ULN)<br>• Elevated Total Bilirubin Levels, (TBL > 1.5 x ULN) and Alkaline Phosphatase, ALP (ALP > 1.5 x ULN), at baseline<br>• Renal impairment at screening (Creatinine clearance [Cockcroft-Gault] <45ml/min)<br>• Acute respiratory failure<br>• Known hyper- or hypothyroidism<br>• History of drug induced Stevens Johnson syndrome, Drug reaction with eosinophilia and systemic symptoms (DRESS syndrome) or toxic epidermal necrolysis or contrast allergy (requiring steroid treatment)<br>• History of recurrent epileptic seizures in the previous 4 years, repetitive or difficult to control seizures, coma or toxic psychosis lasting >48 hours<br>• Average corrected QT interval > 450 msecs using Bazett's formula using triplicate ECGs (or > 480 msecs if bundle branch block)<br>• Known chronic active hepatitis (B or C)<br>• Known HIV infection<br>• Current infection possibly related to recent or on-going immunosuppressive treatment<br>• Known autoimmune disease requiring active immunosuppressive therapy<br>• History of organ transplantation<br>• Any oral or intravenous immunosuppressive treatment including Prednisolone, hydrocortisone or disease modifying drugs such as Azathioprine, interferon-alpha, Cyclophosphamide or Mycophenolate. [Other immunosuppressive therapies should be discussed with PI. Inhaled or topical steroids are permissible.]<br>• Known pregnancy at screening<br>• On-going lactation<br>• Inability to comply with trial procedures<br>• Current participation in the active dosing phase of otherinterventional clinical trials<br>• Contra indication or hypersensitivity to IL-2 treatment or to any of its excipients<br>• Unwillingness or inability to provide written informed consent for participation

For peer review only

		• Any medical history or clinically relevant abnormality that is deemed by the principal investigator/delegate and/or medical monitor to make the patient ineligible for inclusion because of a safety concern

See TIFF Figure 1

Figure 1. Trial design per patient. Each patient will make a total of eight study visits.

See TIFF Figure 2

Figure 2. Trial design for each group in Part A. There are a total of 5 dose levels in Part A.

See TIFF Figure 3

Figure 3. Trial design for each group in Part B. There are a total of 4 dose levels in Part B

Table 2. **Objective stopping criteria triggering a DMC safety review of dose escalation**

QTcB > 500 msecs (or > 530 msecs if baseline QTcB = 450-480 msecs) OR QTcB change from baseline > 60 msecs (based on an average of triplicate ECGs)
Acute Pulmonary oedema or congestive heart failure
Symptomatic systolic BP < 90 mmHg and/or diastolic BP < 60 mmHg OR persistent symptomatic systolic BP 80-90 mmHg for > 15 mins OR severe hypertension (as defined by BP > 180/120 mmHg)
STEMI occurrence
Atrial fibrillation with rapid ventricular response > 150/min, supraventricular tachycardia or bradycardia that requires treatment or is recurrent or persistent
Sustained ventricular tachycardia or ventricular fibrillation
Any patient who develops doubling of creatinine
Systemic hypersensitivity reaction which cannot be attributed to an identifiable cause
If a life-threatening infection is confirmed clinically with a positive microbiological test

For peer review only

For peer review only - http://bmjopen.bmj.com/site/about/guidelines.xhtml

Signs suggestive of hepatic failure including encephalopathy, increasing ascites, signs of coagulopathy, liver pain and/or tenderness on palpation, hypoglycaemia presumed to be secondary to liver failure, active GI bleeding. Withdrawal also if ALT >3 ULN
Seizure activity, coma, severe lethargy or somnolence
Risk of respiratory insufficiency requiring intubation

For peer review only

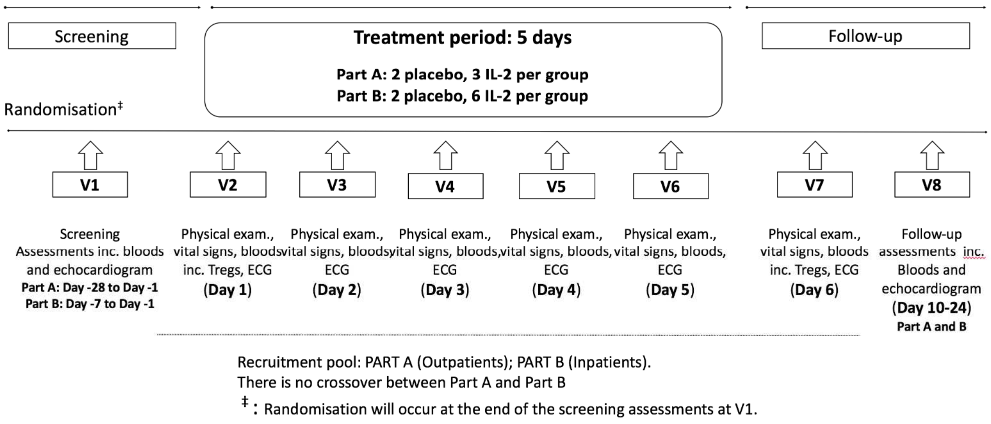

Figure 1. Trial design per patient. Each patient will make a total of eight study visits.

438x188mm (300 x 300 DPI)

For peer review only

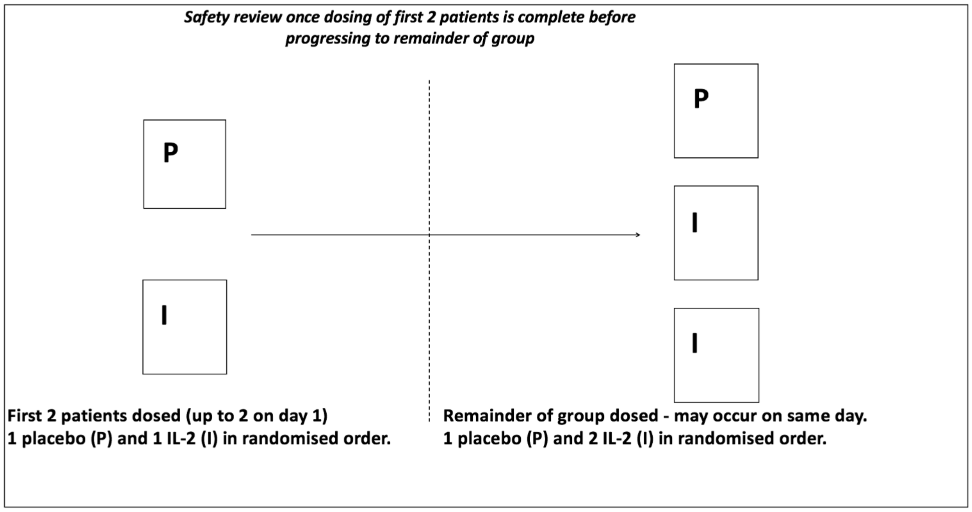

Figure 2. Trial design for each group in Part A. There are a total of 5 dose levels in Part A.

400x214mm (300 x 300 DPI)

review only

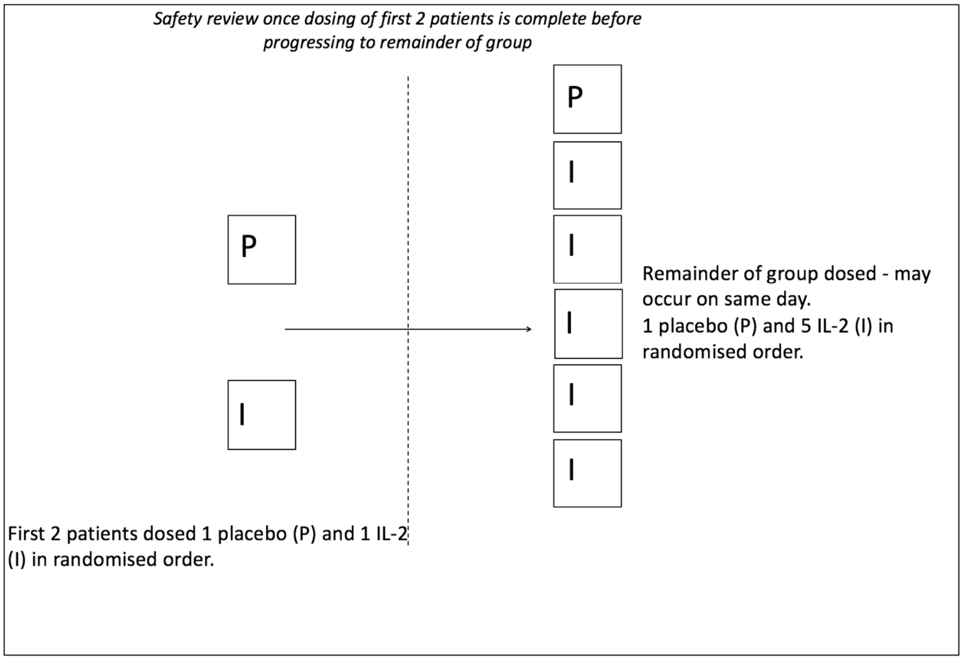

Figure 3. Trial design for each group in Part B. There are a total of 4 dose levels in Part B

329x219mm (300 x 300 DPI)

Supplementary Docuemnt 1

Risk mitigation table.

Potential Risk	Impact - eligibility criteria	Monitoring criteria	Stopping criteria for individual patients
**Cardiac disorders:** Capillary leak syndrome, Cardiac arrhythmias, Transient ECG changes, Angina, Myocardial infarction, Palpitations, Ventricular hypokinesia	Exclusion criteria include: Cardiogenic shock (as defined by systolic blood pressure <80 mmHg unresponsive to fluids or necessitating catecholamines; hypotension (systolic BP < 100 mmHg and/or diastolic BP < 50 mmHg); uncontrolled hypertension (>160/100mmHg); history of recurrent syncope with relevant history suggestive of arrhythmic syncope (e.g. bifascicular block, sinus bradycardia < 40 bpm in the absence of sinoatrial block or medications, preexcited QRS complex, ST segment elevation leads V1 through V3 [Brugada syndrome], negative T wave in the right precordial leads and epsilon wave [arrhythmogenic right ventricular dysplasia/cardiomyopathy], prolonged QT > 450 msecs (or > 480 msecs for patients with bundle branch block); known heart failure with impaired Left ventricular, function (echocardiographically assessed Left ventricular ejection fraction, LVEF < 45% Part A, LVEF < 35% Part B); severe congestive heart failure and/or pulmonary oedema on presentation; ST elevation Myocardial infarction;	On a day-to-day basis vital signs (temperature, blood pressure, heart rate, respiratory rate) will be assessed pre-dosing and every 30 mins (approximately) for 1h post-dosing; 12 lead ECG with QTcB measurement will be performed pre-dosing and approximately 15, 30 mins and at 1 h post-dosing; Continuous cardiac telemetry will be applied during the trial visits for a minimum of 2 hours and up to 6.5 hours. Cardiac biomarkers (TnI and BNP) will be taken prior to dosing (V2) and at the end of active treatment period (V7 and V8). Baseline and post dose echocardiogram will be performed (V1 and V8)	Treatment with the trial drug will be discontinued if: QTcB > 500 msecs (or > 530 msecs if baseline QTcB = 450-480 msecs) OR QTcB change from baseline > 60 msecs (based on an average of triplicate ECGs); New or worsening angina in stable patients (Part A), Worsening angina in ACS patients (Part B); Acute Pulmonary oedema or congestive heart failure; BP stopping criteria: symptomatic systolic BP < 90 and/or diastolic BP < 60 OR persistent symptomatic systolic BP 80-90 mmHg for > 15 mins; OR severe hypertension (as defined by BP > 180/120 mmHg); STEMI occurrence; Atrial fibrillation with rapid ventricular response > 150/min, supraventricular tachycardia or bradycardia that requires treatment or is recurrent or persistent; Sustained ventricular tachycardia or ventricular fibrillation
**Kidney injury or impaired renal function:** Oliguria, Raised serum urea, Raised serum creatinine, Haematuria, Renal failure, Anuria	Part A: Patients with Acute kidney injury (doubling of the serum creatinine from baseline) and/or CKD more than Stage 3B (eGFR = 30-45 ml/min/1.73m^2) will be excluded Part B: Patients with Acute renal impairment at screening (Creatinine clearance [Cockcroft-Gault] <45ml/min) will be excluded	Part A:Kidney function parameters (including serum creatinine, BUN, electrolytes , calcium and eGFR) will be assessed at all visits) Part B:Kidney function parameters (including serum creatinine, BUN, electrolytes and calcium) will be assessed at all visits)	Any patient who develops doubling of creatinine during the trial will be withdrawn from the trial.
**Risk associated with subcutaneous injection of IL-2:** Injection site reaction, pain, inflammation, Mucositis, Injection site nodule, Hypothermia, Injection site necrosis, Erythema,	Patients with a history of known allergy or skin hypersensitivity to IL-2 or any of its excipients will be excluded. History of drug induced Stevens Johnson syndrome, Drug reaction with eosinophilia and systemic symptoms (DRESS syndrome) or toxic epidermal necrolysis.	Injection sites will be examined at each visit and the patients will be assessed for AEs which may be linked to IL-2 related hypersensitivity reactions (erythema, pruritus, angioedema or generalized urticaria).	The investigators should stop the dosing of any patients with any systemic hypersensitivity reaction which cannot be attributed to an identifiable cause. Patients who are dosed and then go on to have a contrast reaction during

For peer review only

Pruritus,<br>Urticaria<br>Malaise, asthenia and fatigue,<br>Pain,<br>Oedema,<br>Weight gain/loss,<br>contrast allergy	Patients with a history of contrast allergy will be excluded from the study in Part B.		this study will be withdrawn from the study
**Infections:**<br>Sepsis<br>Fever with/without chills,	Exclusion criteria include: active infection requiring antibiotic treatment; leukopenia (WBC < 3.3 x 10^3/µL); uncontrolled diabetes (HbA1c > 64 mmol/mol Part A only); current infection possibly related to recent or ongoing immunosuppressive treatment; any oral or intravenous immunosuppressive treatment (including steroids or disease modifying agents such as azathioprine, interferon-alpha, cyclophosphamide, mycophenolate [other immunosuppressive therapies should be discussed with PI]; known HIV infection; known chronic active hepatitis B or C<br><br>Patients with recent infections will only be included when deemed clinically stable by the investigators (when the infection is resolved).	On a day-to-day basis vital signs (temperature, blood pressure, heart rate, respiratory rate) will be assessed pre-dosing and every 30 mins (approximately) for 1h post-dosing<br>Inflammation markers, including WBC + differential as well as CRP will be assessed at baseline and during the treatment period on a daily basis<br>If a patient is found to have pyrexia > 38.5ºC (either in the unit or at home) on 2 separate occasions, diagnostic evaluation (CXR, urine dipstick, blood and urine cultures as directed by symptoms) will be initiated.	If an infection is confirmed clinically with a positive microbiological test, the trial medication will be discontinued.
**Gastrointestinal adverse events:**<br>Nausea with/without vomiting,<br>Diarrhoea,<br>Stomatitis,<br>Dysphagia,<br>Dyspepsia,<br>Constipation,<br>GI bleeding (including rectal haemorrhage, haematemesis),<br>Ascites,<br>Cheilitis,<br>Gastritis,<br>Pancreatitis,<br>Intestinal obstruction,<br>GI perforation,<br>Elevation of hepatic transaminases/ alkaline phosphatase/ lactic dehydrogenase,<br>Hyperbilirubinaemia,<br>Hepatomegaly/ Hepatosplenomegaly<br>Cholecystitis,<br>Liver failure	Exclusion criteria include: known active bleeding (including GI bleeding) or bleeding diatheses; known hepatic failure and/or abnormal LFTs (ALT > 2 x ULN) at baseline; elevated total bilirubin (TBL > 1.5 x ULN) and/or Alkaline Phosphatase levels (ALP > 1.5 x ULN) at baseline; history of chronic active hepatitis B or C;	On a day-to-day basis vital signs, including blood pressure and heart rate will be assessed pre-dosing and every 30 mins (approximately) for 1h post-dosing<br>A daily clinical assessment, including abdominal, skin and mucosal examination will be performed as part of the physical examination.<br>Haemoglobin, haematocrit, platelet counts, BUN, blood glucose, LFTs, TBL and ALP will be assessed at all study visits.	IL-2 treatment should be stopped if the patients show signs suggestive of hepatic failure including encephalopathy, increasing ascites, signs of coagulopathy, liver pain and/or tenderness on palpation, hypoglycaemia presumed to be secondary to liver failure, active GI bleeding. Withdrawal also if ALT >3 ULN
**Neurological events:**<br>Dizziness<br>Headaches<br>Paraesthesia<br>Neuropathy<br>Syncope<br>Speech disorders	Patients with history of recurrent epileptic seizures in the previous 4 years, repetitive or difficult to control seizures, coma or toxic psychosis lasting >48 hours will be excluded.	Changes to the mental status of the trial patients will be monitored during the physical examination for any signs, including moderate confusion or agitation.	The drug will be discontinued in patients who develop seizure activity, coma, severe lethargy or somnolence.

For peer review only

Taste loss<br>Lethargy<br>Coma<br>Convulsions<br>Paralysis<br>Myasthenia<br>Intracranial haemorrhage<br>Cerebrovascular accident<br>Leukoencephalopathy			
**Respiratory events:**<br>Respiratory tract infection,<br>Cough,<br>Dyspnoea,<br>Pulmonary oedema,<br>Pleural effusions,<br>Hypoxia,<br>Haemoptysis,<br>Epistaxis,<br>Nasal congestion,<br>Rhinitis,<br>Pulmonary embolism,<br>Adult respiratory distress syndrome	Patients with a history of underlying respiratory failure, requiring intubation for > 72 hours will be excluded.	Respiratory rates and spO2 will be routinely monitored.	The drug will be discontinued in patients at risk of respiratory insufficiency requiring intubation.
**Embryofetal lethality**<br>Embryofetal studies in rats have shown embryolethality in the presence of maternal toxicity.	Lactating or pregnant female patients will be excluded.	Pregnancy tests for females of child-bearing potential will be performed at screening visit and visit 2 (Part A only) prior to dosing.	Patients who become pregnant during the trial will be withdrawn and followed up appropriately.

For peer review only

Trial Title: Low dose interleukin-2 in patients with stable ischaemic heart disease and acute coronary syndromes (LILACS)

EudraCT No.: 2014-004979-23

IRAS No.: 168647

Trial Sponsor: Cambridge University Hospitals NHS Foundation Trust and University of Cambridge.

Data Monitoring Committee (DMC) Charter

Version 1.1 date 09 NOV 2017
(Developed from DAMOCLES DMC Charter Template v1. February 2005)

Authorised by:

Name: Role:

Signature: Date:

Prepared by:

Name: Role:

Signature: Date:

For peer review only

Table of Contents

CONTENT 3
CHARTER DETAILS 3
1. Introduction 3
Trial Intervention 3
Part A 3
Part B 3
2. Roles and responsibilities 4
3. Before or early in the trial 5
4. Composition 6
5. Relationships 7
6. Organisation of DMC meetings 7
7. Trial documentation and procedures to ensure confidentiality and proper communication 8
8. Decision making 9
9. Reporting 10
10. After the trial 11
Figures and appendices 12
Annex 1: Agreement and potential competing interests form 14
Annex 2: Agreement and confidentiality agreement for observers 15
Annex 3: Suggested report from DMC to TMG where no recommendations are being made 16
Annex 4: LILACS Contacts 17
Annex 5: Summary of changes from previous version 18

For peer review only

For peer review only - http://bmjopen.bmj.com/site/about/guidelines.xhtml

CONTENT	CHARTER DETAILS
**1. INTRODUCTION**	
Name of trial	Low dose interleukin-2 (IL-2) in patients with stable ischaemic heart disease and acute coronary syndromes (LILACS)
Objectives of trial, including interventions being investigated	**TRIAL INTERVENTION**<br>Low-dose IL-2 (aldesleukin)<br><br>**PART A**<br>PRIMARY OBJECTIVES<br>1. Is the administration of IL-2 safe and tolerable in patients with stable ischaemic heart disease?<br><br>Exploratory objectives:<br>1. Does administration of low-dose IL-2 in stable ischaemic heart disease patients result in an increase of circulating Treg levels?<br>2. Does administration of low-dose IL-2 in stable ischaemic heart disease patients result in changes in circulating cardiac biomarkers (including hs-CRP, TnI, IL-6, BNP)?<br>3. Does administration of low-dose IL-2 in stable ischaemic heart disease patients result in changes in lymphocyte subsets?<br>4. Does administration of low-dose IL-2 in stable ischaemic heart disease patients result in changes in circulating IL-2 levels?<br><br>**PART B**<br>Primary objectives<br>1. Does low-dose IL-2 administration in patients with acute coronary syndromes result in an increase mean circulating Treg levels by ≥75% (placebo corrected)?<br>2. Is the administration of IL-2 safe and tolerable in patients with ACS?<br><br>Secondary objectives<br>1. Does administration of low-dose IL-2 in ACS patients result in changes in circulating cardiac biomarkers (including hs-CRP, TnI, IL-6, BNP)?<br>2. Does administration of low-dose IL-2 in ACS patients result in changes in lymphocyte subsets?<br>3. Does administration of low-dose IL-2 in ACS patients result in changes in circulating IL-2 levels?
Outline of scope of charter	The purpose of this document is to describe the membership, terms of reference, roles, responsibilities, authority and decision making of the DMC for the LILACS trial. This includes the timing of meetings, methods of providing information to and from the DMC, frequency and format of meetings, statistical issues and relationships with other committees.

For peer review only

For peer review only - http://bmjopen.bmj.com/site/about/guidelines.xhtml

CONTENT	CHARTER DETAILS
**2. ROLES AND RESPONSIBILITIES**	
A broad statement of the aims of the committee	To protect and serve LILACS trial patients regarding safety and to assist and advise the Chief Investigator and Trial Management Group (TMG) so as to protect the validity and credibility of the trial.<br><br>To safeguard the interests of LILACS patients, assess the safety and efficacy of the interventions during the trial, and monitor the overall conduct of the LILACS trial.
Terms of reference	The DMC should receive and review the progress and accruing data of the LILACS trial and provide advice on the conduct of the trial to the TMG.<br><br>The DMC should inform the Chair of the TMG if, in their view:<br><br>(i) the results are likely to convince a broad range of clinicians, including those supporting the trial and the general clinical community, that one trial arm, or a subset of trial population, is clearly indicated or contraindicated, and there was a reasonable expectation that this new evidence would materially influence patient management; **or**<br><br>(ii) it becomes evident that no clear outcome would be obtained.
Specific roles of DMC	The DMC will review the trial data after completion of Part A of the trial and will provide recommendation for the dose to be used in group B1 (Part B of the trial). The review of the trial's progress will include data quality, and main endpoints including safety data.<br><br>In addition, a DMC meeting might be triggered when:<br><br>Two patients within a trial group experience any combination of: a serious adverse event (SAE) defined as possibly, probably or definitely related to the trial drug (i.e. it is a SAR), an adverse event that is severe and at least possibly related to the trial drug, or any of the objective stopping criteria detailed in the protocol (Any further single instances of the events outlined above for the same group will trigger a DMC safety review). See current protocol for full details of a triggered DMC meeting.<br><br>Specific roles of the DMC include:<br><br>• assess data quality, including completeness and accuracy (and by so doing encourage collection of high quality data)<br>• monitor participant and investigator compliance with the protocol<br>• monitor evidence for treatment differences in the main efficacy endpoints<br>• monitor evidence for treatment harm (eg toxicity data,

For peer review only - http://bmjopen.bmj.com/site/about/guidelines.xhtml

CONTENT	CHARTER DETAILS
	SAEs, deaths)<br>• review all reports of suspected unexpected serious adverse reactions (SUSARs) provided by the trial team<br>• decide whether to recommend that the trial continues to recruit participants or whether recruitment should be terminated either for everyone or for some treatment groups and/or some participant subgroups<br>• suggest additional data analyses<br>• advise on protocol modifications suggested by the TMG (eg inclusion criteria, trial endpoints, or sample size)<br>• monitor continuing appropriateness of patient information<br>• monitor compliance with previous DMC recommendations<br>• consider the ethical implications of any recommendations made by the DMC<br>• assess the impact and relevance of external evidence<br>• maintain confidentiality of all trial information that is not in the public domain<br>• protect validity and scientific credibility of the trial
**3. Before or early in the trial**	
Whether the DMC will have input into the protocol	All potential DMC members should have sight of the protocol/outline before agreeing to join the committee. Before recruitment begins the trial will have undergone review by the funder/sponsor (eg peer review for public sector trials), scrutiny by other trial committees, a research ethics committee (REC), Medicines and Healthcare products Regulatory Agency (MHRA) and Health Research Authority. Therefore, if a potential DMC member has major reservations about the trial (eg the protocol or the logistics) they should report these to the CI or trial coordinating team and may decide not to accept the invitation to join. DMC members should be independent and constructively critical of the ongoing trial, but also supportive of aims and methods of the trial.
Whether the DMC will meet before the start of the trial	It is recommended that, if possible, the DMC meets before the trial starts or early in the course of the trial, to discuss the protocol, the trial, any analysis plan, future meetings, and to have the opportunity to clarify any aspects with the CI(s) and coordinating team. The DMC should meet within one year of recruitment commencing.<br>Consideration should be given to an initial "dummy" report, including the use of shell (empty) tables, to familiarise the DMC members with the format that will be used in the reports.
Any issues specific to the disease under study	The use of IL-2 in cardiovascular patients is currently contraindicated. Part A patients have stable ischaemic heart disease and Part B patients have suffered an Acute

For peer review only

For peer review only - http://bmjopen.bmj.com/site/about/guidelines.xhtml

CONTENT	CHARTER DETAILS
	Coronary Syndrome (ACS).
Any specific regulatory issues	The DMC should be aware of any regulatory implications of their recommendations.
Whether members of the DMC will have a contract	DMC member will not formally sign a contract but should formally register their assent to join the group by confirming (1) that they agree to be on the DMC and (2) that they agree with the contents of this Charter. Any competing interests should be declared at the same time. Members should complete and return the form in Annex 1. All members and observers attending any part of the meeting should sign a confidentiality agreement on the first occasion they attend all or part of a meeting (Annex 2).
**4. Composition**	
Membership and size of the DMC	Membership should consist of a small number of members, who include at least one clinician experienced in the clinical area. Additional members experienced in clinical trials should reflect the other specialities involved in the trial. In the case of intergroup trials or trials with international collaboration consideration should be given to overseas members.<br>The members should not be involved with the trial in any other way or have some competing interest that could impact on the trial. Any competing interests, both real and potential, should be declared. Although members may well be able to act objectively despite such connections, complete disclosure enhances credibility. A short competing interest form should be completed and returned by the DMC members to the trial coordinating team (Annex 1).<br>The members of the DMC for this trial are:<br>(1)<br>(2)<br>(3)<br>(4)
The Chair, how they are chosen and the Chair's role	The Chair should have previous experience of serving on DMCs and experience of chairing meetings, and should be able to facilitate and summarise discussions. The Chair is usually chosen by the CI or TMG or sometimes by the DMC members themselves. The Chair is expected to facilitate and summarise discussions.
The responsibilities of the trial statistician	The trial statistician will have the overall responsibility for producing the report to the DMC and will participate in DMC meetings, guiding the DMC through the report, participating in DMC discussions and, on some occasions, taking notes.
The responsibilities of the trial coordinating team	The trial coordinator/or project manager may help the trial statistician to produce the non-confidential sections of the

For peer review only - http://bmjopen.bmj.com/site/about/guidelines.xhtml

CONTENT	CHARTER DETAILS
	DMC report. The trial coordinator/or project manager may attend open sessions of the meeting.
The responsibilities of the CI and other members of the TMG	The CI, may be asked, and should be available, to attend open sessions of the DMC meeting. The other TMG members will not usually be expected to attend but can attend open sessions when necessary (See Section 6. Organisation of DMC Meetings).
**5. Relationships**	
Relationships with CI(s), other trial committees (TMG), sponsor and regulatory bodies	A diagram is included in this charter (Figure 2) to illustrate the relationships between the trial committees and the sponsor.
Clarification of whether the DMC are advisory (make recommendations) or executive (make decisions)	The TMG will be responsible for the overall supervision of the trial progress, including the choice of doses to give subsequent cohorts of patients. The TMG will meet between trial cohorts within Part A and Part B of the trial and will make executive decisions about the trial during these meetings.<br><br>The DMC will meet between Part A and Part B of the trial to review all data collected in Part A and will determine whether it is safe to progress to Part B of the trial. In addition a DMC meeting may be triggered for safety reasons which are defined in the trial protocol and under 'specific roles of the DMC' in section 2. Under these circumstances the DMC will make executive decisions about the trial.<br><br>If a DMC meeting is convened for reasons other than those described above then their role will be in an advisory capacity to the TMG.
Payments to DMC members	Members will be reimbursed for travel and accommodation where required. No other payments or rewards are given.<br>DMC members should not use interim results to inform trading in pharmaceutical shares, and careful consideration should be given to trading in stock of companies with competing products.
**6. Organisation of DMC meetings**	
Expected frequency of DMC meetings	The exact frequency of meetings will depend upon any statistical plans specified and otherwise on trial events. The wishes of the DMC and needs of the trial coordinating team will be considered when planning each meeting. The DMC should meet at least yearly.<br>An unplanned DMC meeting may be called by the Chair or requested by the TMG if there is an emergency concern on the safety of participants.
Whether meetings will be face-to-face or by teleconference	The first meeting should ideally be face-to-face to facilitate full discussion and allow members to get to know each

For peer review only - http://bmjopen.bmj.com/site/about/guidelines.xhtml

CONTENT	CHARTER DETAILS
	other. If this not possible a video meeting (e.g via skype or GoTomeeting) will be arranged. It is intended that all subsequent meetings should be face-to-face if possible, with teleconference as a second option.
How DMC meetings will be organised, especially regarding open and closed sessions, including who will be present in each session	DMC meetings may contain a mixture of open and closed sessions.<br><br>Closed sessions:<br>Only DMC members and others whom they specifically invite, e.g. the trial statistician, are present in closed sessions.<br>Open sessions:<br>All those attending the closed session may be joined by the CI(s), other members of the trial coordinating team, and sometimes also representatives of the sponsor, funder, or regulator, as relevant.<br><br>Suggested DMC meeting format:<br>1. Open session: Introduction and any "open" parts of the report<br>2. Closed session: DMC discussion of "closed" parts of the report<br>3. Closed session: DMC members private meeting<br>4. Open session: Discussion with other attendees on any matters arising from the previous session(s).<br>5. Closed session: extra closed session as required
**7. TRIAL DOCUMENTATION AND PROCEDURES TO ENSURE CONFIDENTIALITY AND PROPER COMMUNICATION**	
Intended content of material to be available in open sessions	Open sessions: Accumulating information relating to recruitment and data quality (eg data return rates, sample collection) will be presented. Toxicity details based on pooled data will be presented and total numbers of events for the primary outcome measure and other outcome measures may be presented, at the discretion of the DMC.
Intended content of material to be available in closed sessions	Closed sessions: In addition to all the material available in the open session, the closed session material will include efficacy and safety data by treatment group.
Will the DMC be blinded to the treatment allocation	The DMC will not be blinded to the treatment allocation.
The people who will see the accumulating data and interim analysis	The confidential accumulating data and interim analysis by treatment allocation will be seen by the DMC members and the trial statistician(s).<br>DMC members do **not** have the right to share confidential information with anyone outside the DMC, including the CI.
Responsibility for identifying and circulating external evidence (eg from other trials/	Identification and circulation of external evidence (eg from other trials/ systematic reviews) is not the responsibility of the DMC members. The CI, TMG and the trial coordinating team will collate any such information for the presentation

For peer review only

For peer review only - http://bmjopen.bmj.com/site/about/guidelines.xhtml

CONTENT	CHARTER DETAILS
systematic reviews)	in an open session.
To whom the DMC will communicate the decisions/ recommendations that are reached	The DMC should report its decisions / recommendations in writing to the CI and TMG chair. This should be copied to the trial statistician (or trial coordinator) and if possible should be sent via the trial statistician (or trial coordinator) in time for consideration at a TMG meeting where necessary. If the trial is to continue largely unchanged then it is often useful for the report from the DMC to include a summary paragraph suitable for trial promotion purposes. (See Annex 3.)<br>In its communications, the DMC should be careful not to relay any unnecessary information to the TMG.
Whether reports to the DMC be available before the meeting or only at/during the meeting	For planned DMC meetings it is usually helpful for the DMC to receive the report at least 2 weeks before any meetings. For unplanned meetings it may be preferable for all papers to be brought to face-to-face meetings by the trial statistician; time would then be needed for DMC members to assimilate the data/report.
What will happen to the confidential papers after the meeting	The DMC members should store the papers safely after each meeting so they may check the next report against them. After the trial is reported, the DMC members should destroy all interim reports. A copy of all the reports will be held at the Cambridge Clinical Trials Unit.
**8. Decision making**	
What decisions/recommendations will be open to the DMC	Possible decisions/recommendations could include:<br>• No action needed, trial continues as planned<br>• Early stopping due, for example, to clear benefit or harm of a treatment, futility, or external evidence<br>• Stopping recruitment within a subgroup<br>• Extending recruitment or extending follow-up<br>• Sanctioning and/or proposing protocol changes
The role of formal statistical methods, specifically which methods will be used and whether they will be used as guidelines or rules	Interim analyses are scheduled to occur between each group of patients in Part A and Part B of the trial. A report will be generated by the trial statistician that will be reviewed by the TMG who will make decisions about the dose to be used in the next cohort of the trial.<br><br>In addition the DMC will meet to review the safety after Part A of the trial and will decide whether it is safe to progress to Part B of the trial. The minimum dataset required for review between trial groups will be:<br>• All adverse events/adverse reactions<br>• All ECG, blood test results, physical examination reports, echo reports, telemetry summaries and observations up to the V7 time point at a minimum (if not V8)

For peer review only - http://bmjopen.bmj.com/site/about/guidelines.xhtml

CONTENT	CHARTER DETAILS
	• T cell subsets including Tregs and Teffs<br>IL-2 levels would be desirable however not mandatory
How decisions or recommendations will be reached within the DMC	The DMC chair should summarise discussions and encourage consensus; it may be best for the Chair to give their own opinion last. It is important that the implications (e.g. ethical, statistical, practical, financial) for the trial be considered before any recommendation is made<br>It is recommended that every effort should be made for the DMC to reach a unanimous decision. If the DMC cannot achieve this, a vote may be taken, although details of the vote should not be routinely included in the report to the TMG as these may inappropriately convey information about the state of the trial data.
Can DMC members who cannot attend the meeting input	If the report is circulated before the meeting, DMC members who will not be able to attend the meeting may pass comments to the DMC Chair for consideration during the discussions.
What happens to members who do not attend meetings	If a member does not attend a meeting, it should be ensured that the member is available for the next meeting. If a member does not attend a second meeting, they should be asked if they wish to remain part of the DMC.
**9. Reporting**	
To whom will the DMC report their recommendations/decisions, and in what form	This will be a letter to the CI and TMG chair delivered within 3 weeks for planned meetings e.g. for decisions about progression to part B of the trial, and as promptly as possible following unplanned/triggered meetings.<br>A copy of the DMC recommendations/decision letters will be stored in the trial master file.
Whether minutes of the meeting be made and, if so, by whom and where they will be kept	Minutes of the open session will be recorded by a member of the CCTU. Minutes will be finalised upon signature of the chairperson and maintained by the sponsors in accordance with applicable statutory regulations.<br>The minutes of the closed sessions will be recorded by a DMC designee. Minutes from the closed session will be recorded separately from the minutes of the open session and stored securely by the sponsor. Closed session minutes, finalised by signature of the chairperson, will be maintained in confidence and retained until discarded in accordance with applicable statutory regulation.<br>Following each meeting, a report separate from the minutes of the open and closed sessions will be sent to the sponsor/TMG describing the DMC recommendations and rationale for such.
What will be done if there is disagreement between the DMC and the body to which it reports	If the DMC has serious problems or concerns with the TMG decision or vice versa a meeting of these groups should be held. The information to be shown would depend upon the action proposed and the DMC's concerns. Depending on the reason for the disagreement confidential data will often

For peer review only - http://bmjopen.bmj.com/site/about/guidelines.xhtml

CONTENT	CHARTER DETAILS
	have to be revealed to all those attending such a meeting. The meeting should be chaired by a senior member of the CCTU or an external expert who is not directly involved with the trial.
**10. AFTER THE TRIAL**	
Publication of results	At the end of the trial there may be a meeting to allow the DMC to discuss the final data with the key members of TMG and give advice about data interpretation.<br>The DMC may wish to see a statement that the trial results will be published in a correct and timely manner.
The information about the DMC that will be included in published trial reports	DMC members should be named and their affiliations listed in the main report, unless they explicitly request otherwise. A brief summary of the timings and conclusions of DMC meetings should be included in the body of this paper.
Whether the DMC will have the opportunity to approve publications, especially with respect to reporting of any DMC recommendation regarding termination of a trial	The DMC will be given the opportunity to read and comment on publications before submission.
Any constraints on DMC members divulging information about their deliberations after the trial has been published	The DMC may discuss issues from their involvement in the trial 12 months after the primary trial results have been published, or sooner with permission from the TMG.

For peer review only

For peer review only - http://bmjopen.bmj.com/site/about/guidelines.xhtml

FIGURES AND APPENDICES

Figure 1. Trial Flow chart

Figure 2. Relationship of trial committees, including DMC and TMG

Table 1. List of terms

Annex 1: Agreement and potential competing interests form

Annex 2: Agreement and confidentiality agreement for observers

Annex 3: Suggested report from DMC to TMG where no recommendations are being made

Annex 4: Trial Contacts

Annex 5: Summary of changes from previous versions

Figure 1. Trial Flow chart

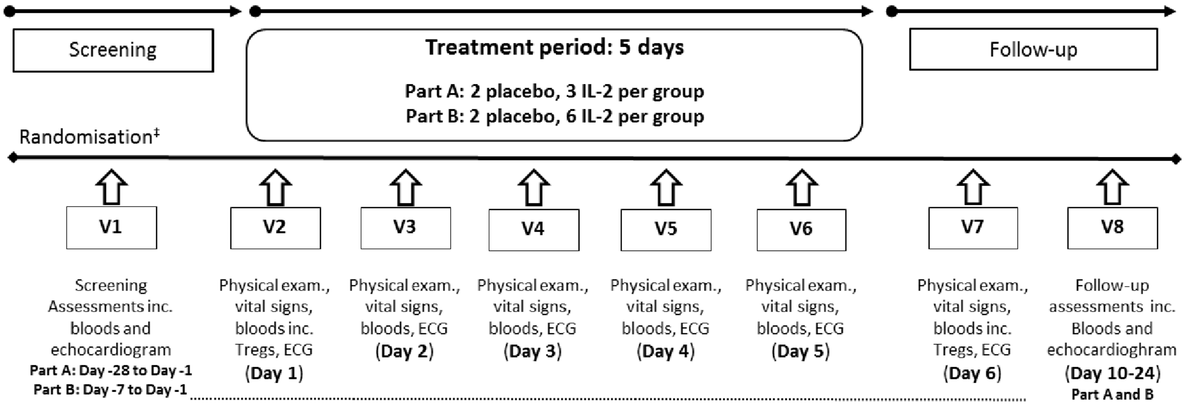

Recruitment pool: PART A (Outpatients); PART B (Inpatients).
There is no crossover between Part A and Part B
‡ : Randomisation will occur at the end of the screening assessments at V1.

Statement of design

This is a repeat-dose, double blind, placebo-controlled, adaptive trial. There are 2 parts of the trial: Part A and Part B, Part B will only begin once Part A has completed. Part A will include in patients with a history of stable ischaemic heart disease, who will be recruited on an outpatient basis, and, Part B will include patients with ACS will be enrolled from an inpatient setting.

For peer review only - http://bmjopen.bmj.com/site/about/guidelines.xhtml

Figure 2. Relationship of trial committees, including, DMC and TMG

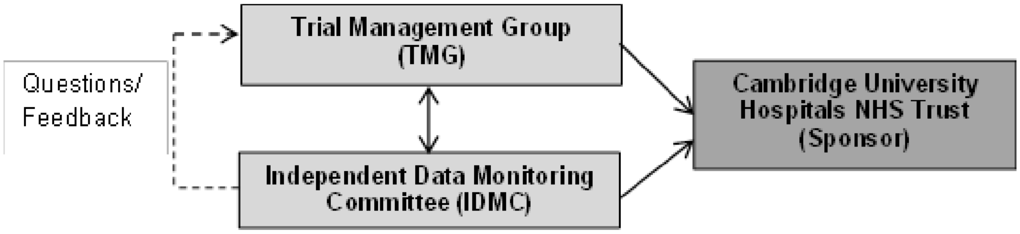

Table 1. List of Terms

Term	Definition
ACS	Acute Coronary Syndrome
AE	Adverse Event
CCTU	Cambridge Clinical Trials Unit
CI	Chief Investigator
CRF	Case Report Form
CTC	Clinical Trials Coordinator
CTIMP	Clinical Trial of an Investigational Medicinal Product
CTM	Clinical Trials Monitor
DM	Data Manager
DMC	Data Monitoring Committee
DMP	Data Management Plan
ID	Identity
HRA	Health Research Authority
IL-2	Interleukin-2
ISF	Investigator Site File
MACRO	Clinical data Management System
MHRA	Medicines Healthcare products Regulatory Agency
PI	Principal Investigator
PID	Patient Identifiable Data
REC	Research Ethics Committee
SAE	Serious Adverse Event
SAR	Serious Adverse Reaction
SDV	Source Data Verification
SUSAR	Suspected Unexpected Serious Adverse Event
Teffs	Effector T cells
TMF	Trial Master File
TMG	Trial Management Group
Tregs	Regulatory T cells

For peer review only - http://bmjopen.bmj.com/site/about/guidelines.xhtml

ANNEX 1: AGREEMENT AND POTENTIAL COMPETING INTERESTS FORM

LILACS

Please complete the following document and return to the LILACS Trial Co-ordinator.

(please initial box to agree)

- ☐ I have read and understood the DMC Charter version 1.0, dated 08 May 2017
- ☐ I agree to join the DMC for this trial
- ☐ I agree to treat all sensitive trial data and discussions confidentially

The avoidance of any perception that members of a DMC may be biased in some fashion is important for the credibility of the decisions made by the DMC and for the integrity of the trial.

Possible competing interest should be disclosed via the Cambridge Clinical Trials Unit (CCTU). In many cases simple disclosure up front should be sufficient. Otherwise, the (potential) DMC member should remove the conflict or stop participating in the DMC. **Table 1** lists potential competing interests.

- ☐ **No,** I have no competing interests to declare
- ☐ **Yes,** I have competing interests to declare (please detail below)

Please provide details of any competing interests:

____

____

____

Name: ______________________________

Signed: ______________________________ Date: ________________

Table 1: Potential competing interests

- Stock ownership in any commercial companies involved
- Stock transaction in any commercial company involved (if previously holding stock)
- Consulting arrangements with the Sponsor (including Chief Investigator for other Cambridge Clinical Trials Unit)
- Frequent speaking engagements on behalf of the intervention
- Career tied up in a product or technique assessed by trial
- Hands-on participation in the trial
- Involvement in the running of the trial
- Emotional involvement in the trial
- Intellectual conflict e.g. strong prior belief in the trial's experimental arm
- Involvement in regulatory issues relevant to the trial procedures
- Investment (financial or intellectual) or career tied up in competing products
- Involvement in the publication in the form of authorship

For peer review only

For peer review only - http://bmjopen.bmj.com/site/about/guidelines.xhtml

Annex 2: Agreement and confidentiality agreement for observers

LILACS

Please complete the following document and return to the LILACS Co-ordinator.

(please initial box to agree)

	I have received a copy of the DMC Charter version 1.1, dated 09 Nov 2017
	I agree to attend the DMC meeting on ___/____/______
	I agree to treat as confidential any sensitive trial information gained during this meeting unless explicitly permitted

Name: ______________________________

Signed: _____________________________ Date: _______________

For peer review only - http://bmjopen.bmj.com/site/about/guidelines.xhtml

ANNEX 3: SUGGESTED REPORT FROM DMC TO TMG WHERE NO RECOMMENDATIONS ARE BEING MADE

[Insert date]

To: Chair of Trial Management Group
Via: Trial statistician or Trial co-ordinator

Dear *[Chair of Trial Management Group]*

The Independent Data Monitoring Committee (DMC) for the LILACS met on *[meeting date]* to review its progress and interim accumulating data. *[List members] attended* the meeting and reviewed the report.

The DMC should like to congratulate the investigators and trial team on the running of the trial and its recruitment, data quality and follow-up. The trial question remains important and, on the basis of the data reviewed at this stage, we recommend continuation of the trial according to the current version of the protocol *[specify protocol version number and date]* with no changes.

We shall next review the progress and data *[provide approximate timing]*

Yours sincerely,

Name of the Chair,

On behalf of the DMC (all members listed below)

DMC members:
(1)
(2)
(3)
(4)

For peer review only

For peer review only - http://bmjopen.bmj.com/site/about/guidelines.xhtml

ANNEX 4: LILACS CONTACTS

For peer review only

For peer review only - http://bmjopen.bmj.com/site/about/guidelines.xhtml

ANNEX 5: SUMMARY OF CHANGES FROM PREVIOUS VERSION

Version 1.1 Dated 9 Nov 2017: Figure 1 updated and name of Data Manager added.

For peer review only

For peer review only - http://bmjopen.bmj.com/site/about/guidelines.xhtml

SPIRIT
STANDARD PROTOCOL ITEMS: RECOMMENDATIONS FOR INTERVENTIONAL TRIALS

SPIRIT 2013 Checklist: Recommended items to address in a clinical trial protocol and related documents*

Section/item	Item No	Description
**Administrative information**		
Title	1	Descriptive title identifying the study design, population, interventions, and, if applicable, trial acronym<br>Page 1
Trial registration	2a	Trial identifier and registry name. If not yet registered, name of intended registry<br>Page 2
	2b	All items from the World Health Organization Trial Registration Data Set<br>Supp 3
Protocol version	3	Date and version identifier<br>Supp 3
Funding	4	Sources and types of financial, material, and other support<br>Supp 3, page 11
Roles and responsibilities	5a	Names, affiliations, and roles of protocol contributors<br>Page 1, 12
	5b	Name and contact information for the trial sponsor<br>Supp 3
	5c	Role of study sponsor and funders, if any, in study design; collection, management, analysis, and interpretation of data; writing of the report; and the decision to submit the report for publication, including whether they will have ultimate authority over any of these activities<br>Page 11
	5d	Composition, roles, and responsibilities of the coordinating centre, steering committee, endpoint adjudication committee, data management team, and other individuals or groups overseeing the trial, if applicable (see Item 21a for data monitoring committee)<br>Pages 7,8,10, 12, Suppl 2
**Introduction**		

For peer review only

Background and rationale	6a	Description of research question and justification for undertaking the trial, including summary of relevant studies (published and unpublished) examining benefits and harms for each intervention<br>Page 3
	6b	Explanation for choice of comparators<br>Pages 5,6
Objectives	7	Specific objectives or hypotheses<br>Page 5
Trial design	8	Description of trial design including type of trial (eg, parallel group, crossover, factorial, single group), allocation ratio, and framework (eg, superiority, equivalence, noninferiority, exploratory)<br>Page 5
**Methods: Participants, interventions, and outcomes**		
Study setting	9	Description of study settings (eg, community clinic, academic hospital) and list of countries where data will be collected. Reference to where list of study sites can be obtained<br>Page 5,6
Eligibility criteria	10	Inclusion and exclusion criteria for participants. If applicable, eligibility criteria for study centres and individuals who will perform the interventions (eg, surgeons, psychotherapists)<br>Pages 5,6, 15, 16, 17
Interventions	11a	Interventions for each group with sufficient detail to allow replication, including how and when they will be administered<br>Page 6
	11b	Criteria for discontinuing or modifying allocated interventions for a given trial participant (eg, drug dose change in response to harms, participant request, or improving/worsening disease)<br>Page 10, table 2
	11c	Strategies to improve adherence to intervention protocols, and any procedures for monitoring adherence (eg, drug tablet return, laboratory tests)<br>n/a
	11d	Relevant concomitant care and interventions that are permitted or prohibited during the trial<br>Pages 15,16, 17

Outcomes	12	Primary, secondary, and other outcomes, including the specific measurement variable (eg, systolic blood pressure), analysis metric (eg, change from baseline, final value, time to event), method of aggregation (eg, median, proportion), and time point for each outcome. Explanation of the clinical relevance of chosen efficacy and harm outcomes is strongly recommended<br>Pages 8,9
Participant timeline	13	Time schedule of enrolment, interventions (including any run-ins and washouts), assessments, and visits for participants. A schematic diagram is highly recommended (see Figure)<br>Figure 1,2,3
Sample size	14	Estimated number of participants needed to achieve study objectives and how it was determined, including clinical and statistical assumptions supporting any sample size calculations<br>Page 10
Recruitment	15	Strategies for achieving adequate participant enrolment to reach target sample size<br>Page 7

Methods: Assignment of interventions (for controlled trials)

Allocation:

Sequence generation	16a	Method of generating the allocation sequence (eg, computer-generated random numbers), and list of any factors for stratification. To reduce predictability of a random sequence, details of any planned restriction (eg, blocking) should be provided in a separate document that is unavailable to those who enrol participants or assign interventions<br>Pages 6, 7
Allocation concealment mechanism	16b	Mechanism of implementing the allocation sequence (eg, central telephone; sequentially numbered, opaque, sealed envelopes), describing any steps to conceal the sequence until interventions are assigned<br>Page 6
Implementatio n	16c	Who will generate the allocation sequence, who will enrol participants, and who will assign participants to interventions<br>Page 6
Blinding (masking)	17a	Who will be blinded after assignment to interventions (eg, trial participants, care providers, outcome assessors, data analysts), and how<br>Pages 5,6,7

	17b	If blinded, circumstances under which unblinding is permissible, and procedure for revealing a participant's allocated intervention during the trial<br>Page 6

Methods: Data collection, management, and analysis

Data collection methods	18a	Plans for assessment and collection of outcome, baseline, and other trial data, including any related processes to promote data quality (eg, duplicate measurements, training of assessors) and a description of study instruments (eg, questionnaires, laboratory tests) along with their reliability and validity, if known. Reference to where data collection forms can be found, if not in the protocol<br>Pages 9, 10, 11
	18b	Plans to promote participant retention and complete follow-up, including list of any outcome data to be collected for participants who discontinue or deviate from intervention protocols<br>n/a
Data management	19	Plans for data entry, coding, security, and storage, including any related processes to promote data quality (eg, double data entry; range checks for data values). Reference to where details of data management procedures can be found, if not in the protocol<br>Page 11
Statistical methods	20a	Statistical methods for analysing primary and secondary outcomes. Reference to where other details of the statistical analysis plan can be found, if not in the protocol<br>Page 10, 11
	20b	Methods for any additional analyses (eg, subgroup and adjusted analyses)<br>n/a
	20c	Definition of analysis population relating to protocol non-adherence (eg, as randomised analysis), and any statistical methods to handle missing data (eg, multiple imputation)<br>n/a

Methods: Monitoring

Data monitoring	21a	Composition of data monitoring committee (DMC); summary of its role and reporting structure; statement of whether it is independent from the sponsor and competing interests; and reference to where further details about its charter can be found, if not in the protocol. Alternatively, an explanation of why a DMC is not needed<br>Page 7,10, Supp 2

	21b	Description of any interim analyses and stopping guidelines, including who will have access to these interim results and make the final decision to terminate the trial<br>Page 10, Table 2
Harms	22	Plans for collecting, assessing, reporting, and managing solicited and spontaneously reported adverse events and other unintended effects of trial interventions or trial conduct<br>Page 8
Auditing	23	Frequency and procedures for auditing trial conduct, if any, and whether the process will be independent from investigators and the sponsor<br>n/a
**Ethics and dissemination**		
Research ethics approval	24	Plans for seeking research ethics committee/institutional review board (REC/IRB) approval<br>Page 11
Protocol amendments	25	Plans for communicating important protocol modifications (eg, changes to eligibility criteria, outcomes, analyses) to relevant parties (eg, investigators, REC/IRBs, trial participants, trial registries, journals, regulators)<br>n/a
Consent or assent	26a	Who will obtain informed consent or assent from potential trial participants or authorised surrogates, and how (see Item 32)<br>Pages 6,7
	26b	Additional consent provisions for collection and use of participant data and biological specimens in ancillary studies, if applicable<br>n/a
Confidentiality	27	How personal information about potential and enrolled participants will be collected, shared, and maintained in order to protect confidentiality before, during, and after the trial<br>Page 11
Declaration of interests	28	Financial and other competing interests for principal investigators for the overall trial and each study site<br>Page 12
Access to data	29	Statement of who will have access to the final trial dataset, and disclosure of contractual agreements that limit such access for investigators<br>n/a

For peer review only

Ancillary and post-trial care	30	Provisions, if any, for ancillary and post-trial care, and for compensation to those who suffer harm from trial participation<br>n/a
Dissemination policy	31a	Plans for investigators and sponsor to communicate trial results to participants, healthcare professionals, the public, and other relevant groups (eg, via publication, reporting in results databases, or other data sharing arrangements), including any publication restrictions<br>Page 11
	31b	Authorship eligibility guidelines and any intended use of professional writers<br>n/a
	31c	Plans, if any, for granting public access to the full protocol, participant-level dataset, and statistical code<br>n/a
**Appendices**		
Informed consent materials	32	Model consent form and other related documentation given to participants and authorised surrogates<br>n/a
Biological specimens	33	Plans for collection, laboratory evaluation, and storage of biological specimens for genetic or molecular analysis in the current trial and for future use in ancillary studies, if applicable<br>Page 9, 10

*It is strongly recommended that this checklist be read in conjunction with the SPIRIT 2013 Explanation & Elaboration for important clarification on the items. Amendments to the protocol should be tracked and dated. The SPIRIT checklist is copyrighted by the SPIRIT Group under the Creative Commons "Attribution-NonCommercial-NoDerivs 3.0 Unported" license.

For peer review only
